# Dinuclear nitrido-bridged osmium complexes inhibit the mitochondrial calcium uniporter and protect cortical neurons against lethal oxygen–glucose deprivation†

**DOI:** 10.1039/d2cb00189f

**Published:** 2022-11-14

**Authors:** Joshua J. Woods, Robyn J. Novorolsky, Nicholas P. Bigham, George S. Robertson, Justin J. Wilson

**Affiliations:** a Department of Chemistry and Chemical Biology, Cornell University Ithaca NY 14853 USA jjw275@cornell.edu; b Robert F. Smith School for Chemical and Biomolecular Engineering, Cornell University Ithaca NY 14853 USA; c Department of Pharmacology, Faculty of Medicine, Dalhousie University, Life Sciences Research Institute Halifax NS B3H 0A8 Canada George.Robertson@Dal.Ca; d Brain Repair Centre, Faculty of Medicine, Dalhousie University, Life Sciences Research Institute Halifax NS B3H 0A8 Canada; e Department of Psychiatry, Faculty of Medicine, Dalhousie University, Life Sciences Research Institute Halifax NS B3H0A8 Canada

## Abstract

Dysregulation of mitochondrial calcium uptake mediated by the mitochondrial calcium uniporter (MCU) is implicated in several pathophysiological conditions. Dinuclear ruthenium complexes are effective inhibitors of the MCU and have been leveraged as both tools to study mitochondrial calcium dynamics and potential therapeutic agents. In this study, we report the synthesis and characterization of Os245 ([Os_2_(μ-N)(NH_3_)_8_Cl_2_]^3+^) which is the osmium-containing analogue of our previously reported ruthenium-based inhibitor Ru265. This complex and its aqua-capped analogue Os245′ ([Os_2_(μ-N)(NH_3_)_8_(OH_2_)_2_]^5+^) are both effective inhibitors of the MCU in permeabilized and intact cells. In comparison to the ruthenium-based inhibitor Ru265 (*k*_obs_ = 4.92 × 10^−3^ s^−1^), the axial ligand exchange kinetics of Os245 are two orders of magnitude slower (*k*_obs_ = 1.63 × 10^−5^ s^−1^) at 37 °C. The MCU-inhibitory properties of Os245 and Os245′ are different (Os245 IC_50_ for MCU inhibition = 103 nM; Os245′ IC_50_ for MCU inhibition = 2.3 nM), indicating that the axial ligands play an important role in their interactions with this channel. We further show that inhibition of the MCU by these complexes protects primary cortical neurons against lethal oxygen–glucose deprivation. When administered *in vivo* to mice (10 mg kg^−1^), Os245 and Os245′ induce seizure-like behaviors in a manner similar to the ruthenium-based inhibitors. However, the onset of these seizures is delayed, a possible consequence of the slower ligand substitution kinetics for these osmium complexes. These findings support previous studies that demonstrate inhibition of the MCU is a promising therapeutic strategy for the treatment of ischemic stroke, but also highlight the need for improved drug delivery strategies to mitigate the pro-convulsant effects of this class of complexes before they can be implemented as therapeutic agents. Furthermore, the slower ligand substitution kinetics of the osmium analogues may afford new strategies for the development and modification of this class of MCU inhibitors.

## Abstract 2

We have investigated the biological properties of the osmium analogue of a potent ruthenium-based mitochondrial calcium uniporter inhibitor and have found it to possess distinct properties.

## Graphical abstract

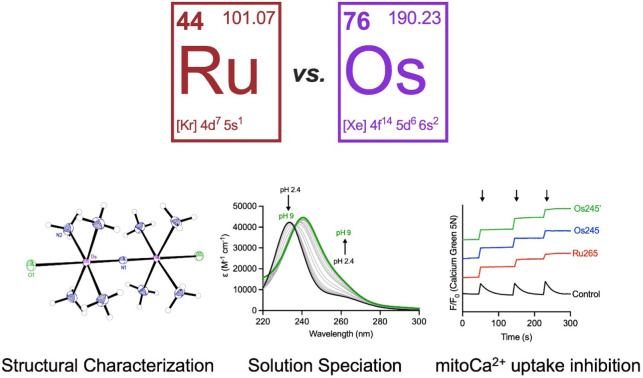

## Introduction

**Scheme 1:**
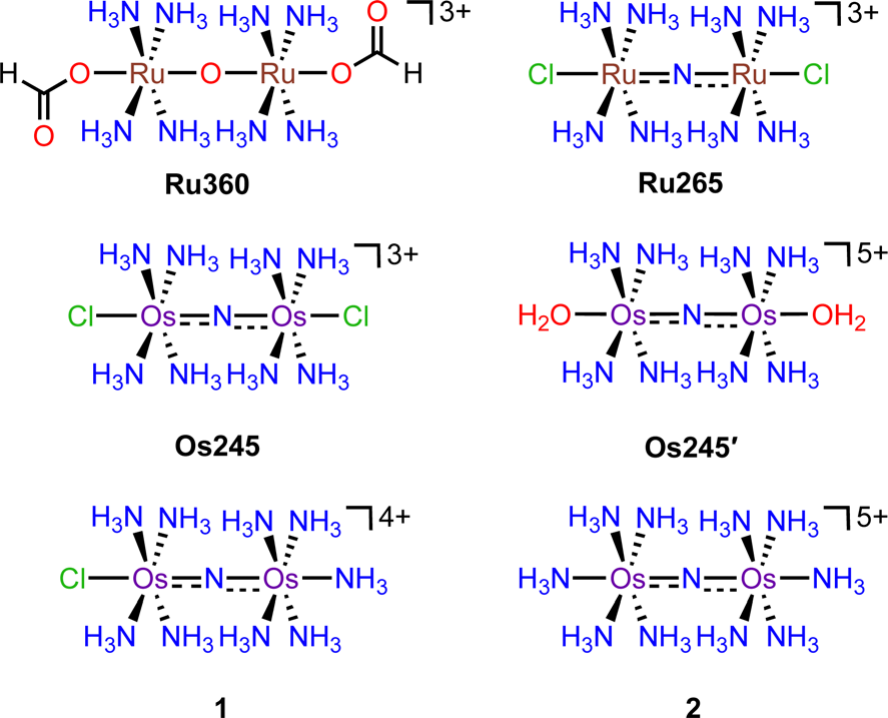
Chemical structures of the complexes discussed in this work.

Critical biological processes, including the production of adenosine triphosphate (ATP) *via* oxidative phosphorylation, hormone and porphyrin synthesis, and lipid metabolism, occur within the mitochondria of eukaryotic cells. The regulation of these processes is mediated in part by mitochondrial Ca^2+^ (mt-Ca^2+^) uptake, which shapes cytosolic Ca^2+^ dynamics^1^ and stimulates matrix dehydrogenases that produce reducing equivalents which drive oxidative phosphorylation. Transport of Ca^2+^ into the mitochondria occurs *via* the mitochondrial calcium uniporter (MCU) complex.^2^ This protein is composed of the pore-forming MCU subunit and the regulatory EMRE, MICU1, and MICU2 subunits.^3–5^ Dysregulation of mt-Ca^2+^ uptake by the MCU can lead to mt-Ca^2+^ overload and cell death, which has been implicated in neuromuscular disease, cancer, and ischemic reperfusion injury.^6,7^ The role of mt-Ca^2+^ uptake in such pathologies has prompted the search for inhibitors of the MCU as both tools to understand the mechanisms of mt-Ca^2+^ dynamics and as potential therapeutic agents.^8–10^ This work has led to the discovery of several organic^11–16^ and inorganic^17–22^ small molecules that can modulate mt-Ca^2+^ uptake through the MCU. The most commonly used MCU inhibitor is the dinuclear oxo-bridged ruthenium complex Ru360, which is named for its strong absorbance at 360 nm (Scheme 1).^17,23,24^ Although this complex is a highly potent inhibitor of the MCU,^17^ its widespread application in biology is hampered by its poor cell permeability^17,25,26^ and instability towards reduction.^21^ Furthermore, Ru360 is of limited commercial availability and is somewhat challenging to synthesize, purify, and characterize.^18^ To address the deficiencies of Ru360, we have been developing alternative coordination complexes as tools for regulating mt-Ca^2+^ uptake in biological systems.^18–21,27–29^ This work led to the discovery of the nitrido-bridged diruthenium complex Ru265 (Scheme 1), which inhibits MCU-mediated mt-Ca^2+^ uptake in intact cells^19,20^ and protects against mt-Ca^2+^ overload in an *in vivo* model of ischemic stroke.^27^ Further studies have also used this complex to investigate the role of the MCU in cancer cell death,^30^ ameloblast Ca^2+^ signaling,^31^ and astrocyte function.^32^

In continuing our study of Ru265, we sought to understand the influence of the metal center on the biological properties of this compound. To this end, we have synthesized the nitrido-bridged diosmium complex [Os_2_(μ-N)(NH_3_)_8_Cl_2_]^3+^ (Os245), which is a structural analogue of Ru265 (Scheme 1). We have thoroughly explored the physical properties of this complex and its analogue Os245′ (Scheme 1) where the axial chlorido ligands have been replaced with coordinated water molecules. Cellular accumulation and mt-Ca^2+^ uptake studies demonstrate that both complexes are cell-permeable and can inhibit mt-Ca^2+^ uptake in permeabilized and intact cells. We further investigated the ability of Os245 and Os245′ to protect cortical neurons against oxygen–glucose deprivation (OGD), an *in vitro* model for ischemic stroke. Both complexes preserve mitochondrial bioenergetics in cortical neurons exposed to a lethal period of OGD. Lastly, we show that, like Ru265,^27^ Os245 and Os245′ induce seizure-like behavior in mice at a dose of 10 mg kg^−1^. However, this response is delayed for the Os complexes compared to Ru265, which may be a result of the increased inertness of Os compared to Ru. Taken together, this work highlights how the identity of the metal center and axial ligands can influence the biological activity of this class of MCU inhibitors.

## Results and discussion

Previous reports describe the synthesis of Os245 by heating M_2_OsCl_6_ (M = Na, K) or K_3_[Os_2_(μ-N)Cl_8_(OH_2_)_2_] and concentrated NH_4_OH in a pressurized tube.^33–35^ Because the synthesis of [Os_2_(μ-N)Cl_8_(OH_2_)_2_]^3−^ requires the harsh conditions of heating (NH_4_)_2_OsCl_6_ under a current of dried Cl_2_ gas at 400 °C,^33^ we first attempted to access Os245 by treating commercially available (NH_4_)_2_OsCl_6_ with concentrated NH_4_OH in a sealed pressure vessel. Under these conditions we obtained a microcrystalline yellow solid that consisted of a mixture of three major species, which were identified by ^1^H NMR spectroscopy to be [Os_2_(μ-N)(NH_3_)_9_Cl]Cl_4_ (1), [Os_2_(μ-N)(NH_3_)_10_]Cl_5_ (2), and Os245 (Scheme 1 and Fig. S1, ESI†). These results agree with previous studies that found the reaction between Na_2_OsCl_6_ and NH_4_OH yields a complex mixture of products.^34,35^ Notably, similar mixtures of products were obtained when Na_2_OsCl_6_ or K_2_OsCl_6_ were used as the source of Os (Table S1, ESI†), suggesting that the additional NH_4_^+^ counterions present in (NH_4_)_2_OsCl_6_ do not significantly influence the outcome of the reaction. All three complexes were obtained when the reaction was allowed to proceed for 12, 48, or 72 h and when 7 M NH_4_OH was used in place of 14 M NH_4_OH (Table S1, ESI†). Given that 1, 2, and Os245 display highly similar infrared and electronic absorption spectra,^33–36^ it is possible that the early literature reports of Os245, which did not characterize the complexes by NMR spectroscopy, actually describe the isolation of this mixture of species, rather than a single pure compound. It should also be mentioned that the attempted synthesis of [Os_2_(μ-N)Cl_8_(OH_2_)_2_]^3−^*via* reduction of [OsCl_5_(NO)]^2−^ with SnCl_2_ in boiling HCl, the method used for the synthesis of [Ru_2_(μ-N)Cl_8_(OH_2_)_2_]^3−^,^20,33^ was unsuccessful and yielded [OsCl_5_(NH_3_)]^2−^ as the only isolable species.

**Fig. 1:**
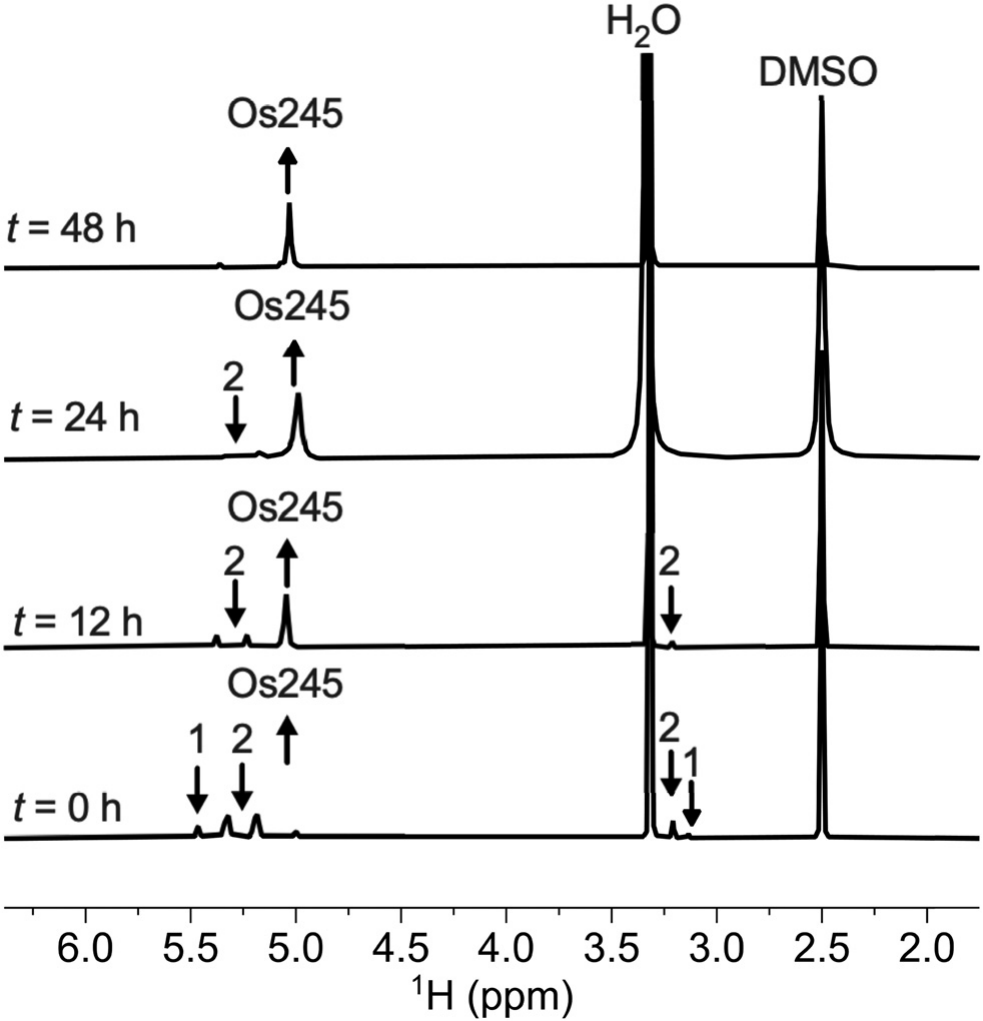
^1^H NMR spectra (500 MHz, 25 °C, DMSO-*d*_6_) showing the conversion of 1 and 2 to Os245 by heating in refluxing HCl. Arrows indicate the growth or disappearance of peaks.

As noted above, the complexes 1, 2, and Os245 are chemically similar, a property that renders their separation to be challenging. The axial ligand substitution differences (Cl^−^*vs.* NH_3_), however, manifests in a different overall complex charge (+3, +4 and +5) among the three complexes. As such, we reasoned that ion exchange chromatography would provide an effective means for their separation. Unfortunately, all three complexes coeluted with 3–4 M HCl when using DOWEX 50W-X2 cation-exchange resin. Other resins that were tested, Sephadex G25, SP-Sephadex-C25, CM-Sephadex, and CM-cellulose, also failed to afford an efficient separation of the three species. Our successful isolation of Os245 was inspired by the observation that axial ammine ligands of 2 are somewhat labile.^36^ We found that mixtures containing Os245, 1, and 2 could be converted to pure Os245 by heating the mixed product at reflux in 4 M HCl for several days (Fig. 1). With pure Os245 in hand, its diaqua-capped analogue Os245′ (Scheme 1) was obtained by treating Os245 with 5 equivalents of AgOTf (OTf^−^ = trifluoromethanesulfonate) at 50 °C in water to remove the chlorido ions as insoluble AgCl.

Os245 and Os245′ were characterized by standard techniques, including IR, UV-vis, and NMR spectroscopies as well as cyclic voltammetry (Table 1). Like Ru265, the electrons within the two d^4^ Os^4+^ centers are strongly coupled, making the complex diamagnetic and amenable to characterization by NMR spectroscopy. In DMSO-*d*_6_, the symmetry-equivalent protons of the equatorial ammine ligands in Os245 and Os245′ appear as a relatively sharp singlet at 5.03 ppm and 4.83 ppm, respectively (Fig. S2–S4, ESI†). The IR spectra of the complexes feature sharp bands near 1108 cm^−1^ (Os245) and 1124 cm^−1^ (Os245′), which correspond to the asymmetric Os–N–Os stretching mode (Fig. S5 and S6, ESI†). The blue-shift of this stretching mode compared to Ru265 (1050 cm^−1^) suggests stronger π-bonding in the Os–N–Os moiety compared to the isostructural Ru analogue. The electronic absorption spectrum of Os245 in 50 mM MOPS (pH 7.4) consists of a strong charge-transfer band centered at 245 nm (*ε* = 50 000 ± 2600 M^−1^ cm^−1^), which forms the basis of the name of this compound, and a lower energy shoulder at 276 nm (*ε* = 7700 ± 360 M^−1^ cm^−1^; Fig. S7, ESI†). The spectrum of Os245′ displays a similar band at 239 nm (Fig. S8, ESI†). Cyclic voltammetry of Os245 in 0.1 M KCl buffered to pH 7.4 with 10 mM HEPES shows that the complex does not undergo any reduction or oxidation events within the window of −1 to 1 V (*vs.* SCE), indicating that it is redox inert within this range like Ru265 (Fig. S9, ESI†).^21^ Under these same conditions, Os245′ shows an irreversible oxidation at 685 mV *vs.* SCE, which is assigned to the (Os^4+^,Os^4+^)/(Os^4+^,Os^5+^) redox couple.^34^ The lower oxidation potential of Os245′ compared to Os245 is consistent with Lever's electrochemical parameters (*E*_L_) for Cl^−^ (−0.24) and OH^−^ (−0.59),^37^ with the parameter for hydroxido being chosen due to the aqueous speciation of Os245 (*vide infra*). Given that most biological reducing agents possess redox potentials within the range of −0.744 V to +0.556 V *vs.* SCE, it is unlikely that this oxidation event will play a significant role in the biological activity of these complexes.

**Table 1:** Comparison of relevant physical properties of Ru265, Os245, Ru265′ and Os245′ a

Property	Ru265	Os245	Ru265′	Os245′
*λ*_max_, nm (*ε* × 10^−4^, M^−1^ cm^−1^)	265 (3.4 ± 0.2),	245 (5 ± 0.26),	nd^b^	240 (4.1 ± 0.1)
	322 (1.4 ± 0.12)	276 (0.77 ± 0.04)		
*ν*_M–N–M_, cm^−1^	1050	1108	nd	1124
M–N–M angle, °	180	180	180	180
Aquation *k*_obs_, s^−1^ (50 mM MOPS, pH 7.4, 37 °C)	(4.92 ± 0.66) × 10^−3^	(1.63 ± 0.24) × 10^−5^	—	—
Aquation *t*_1/2_, min (50 mM MOPS, pH 7.4, 37 °C)	2.3	700.1	—	—
Aquation *k*_obs_, s^−1^ (50 mM MOPS + 150 mM NaCl, pH 7.4, 37 °C)	(5.41 ± 0.20) × 10^−3^	(1.68 ± 0.14) × 10^−5^	—	—
Aquation *t*_1/2_, min (50 mM MOPS + 150 mM NaCl, pH 7.4, 37 °C)	2.1	687.6	—	—
p*K*_a1_	—	—	5.14(02)	4.77(01)
p*K*_a2_	—	—	7.00(11)	6.41(09)
Charge at pH 7.4	+3	+3	+3/+4^c^	+3/+4^d^
Redox events, mV *vs.* SCE^e^	—	—	—	685

a Data for Ru265 and Ru265′ taken from ref. 20 and 21.

b Not determined.

c These species exist in a ratio of 72 : 28.

d These species exist in a ratio of 92 : 8.

e 0.1 M KCl + 10 mM HEPES (pH 7.4, 23 °C).

**Fig. 2:**
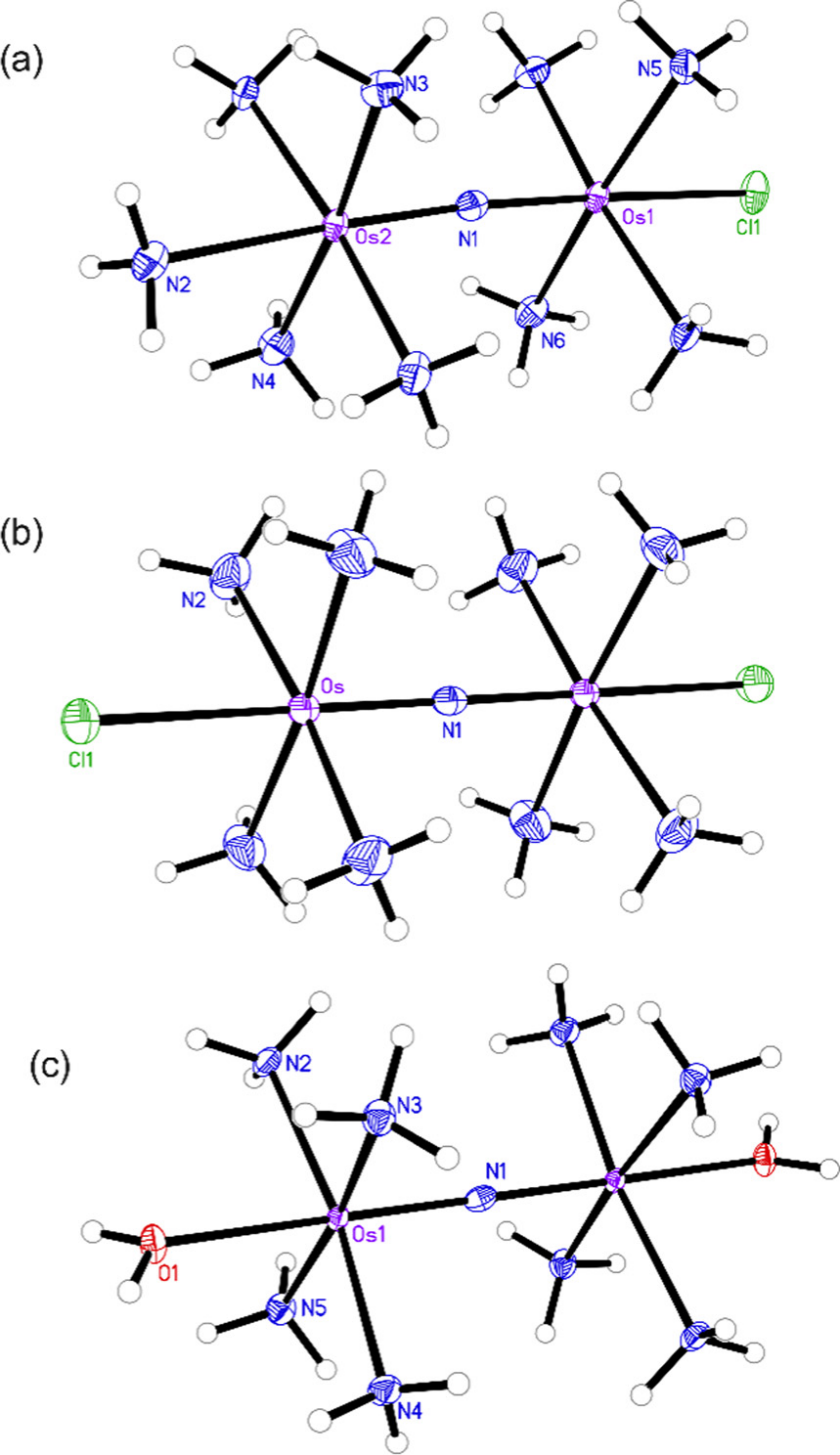
X-ray crystal structures of (a) compound 1, (b) Os245, and (c) Os245′. Outer-sphere solvent molecules and counterions are omitted for clarity. Ellipsoids are drawn at the 50% probability level. For Os245′, only one of the two independent molecules present in the asymmetric unit is shown. Unlabeled atoms are symmetry generated. Selected geometric parameters are given in Tables S2–S5 (ESI†).

The structures of the byproduct 1, Os245, and Os245′ were further elucidated by single-crystal X-ray diffraction (Fig. 2). Details of the crystal structure refinement and relevant interatomic distances and angles are reported in Tables S2–S5 (ESI†). The crystal structures confirm both the identity of the axial ligands assigned by NMR spectroscopy and the linear Os–N–Os motif (Table 1). As for Ru265 and Ru265′,^20,21^ the equatorial ammine ligands of the bridged Os^4+^ centers are arranged in an eclipsed configuration for all three complexes. The Os–N_nitrido_ distances, 1.7688(3), 1.76340(9), and 1.759(5) Å for Os245, Os245′, and 1 respectively, agree well with previously reported nitrido-bridged Os compounds.^38–41^ In the structure of 1, the interatomic distance between the osmium center and the ammine ligand in the axial coordination site (N2 in Fig. 2a) is approximately 0.07 Å longer than those the in equatorial positions, which is due to the trans influence of the nitrido bridge.

Like Ru265,^21^ Os245 undergoes aquation in water to afford the diaqua complex Os245′. The aquation of Os245 in 50 mM MOPS (pH 7.4) at 37 °C was monitored by UV-vis spectroscopy by following the change in absorbance at 245 nm (Fig. S10, ESI†). As with Ru265, our kinetic analysis using this technique showed a simple mono-exponential decay of the starting material, which we hypothesize arises from the indistinguishably similar UV-vis spectra of the mono and disubstituted reaction products.^29^ As such, the aquation reaction was treated as a single-step, pseudo-first-order reaction. The obtained *k*_obs_ value for the aquation of Os245 at 37 °C is two orders of magnitude smaller than that of Ru265 (Table 1) and corresponds to a half-life of approximately 12 h. These results are consistent with the greater inertness of Os complexes compared to their isostructural Ru analogues.^42^ The slow substitution kinetics of the axial ligands may enable them to be retained on biologically relevant time scales and could therefore affect the biological activities of the resulting Os complexes. This property contrasts with the faster-substituting Ru analogue, where the aquated product forms rapidly upon dissolution in water and is the predominate species in biological solutions. To assess the role of the high chloride ion concentrations found extracellularly, these aquation studies were also carried out in the presence of 150 mM NaCl (Table 1 and Fig. S11, ESI†). Notably, the rate constant is not significantly different from the chloride-free conditions. This result is consistent with that previously observed for Ru265.

Water molecules that are coordinated to metal ions are generally acidic, and the p*K*_a_ values of these ligands dictate the speciation of the metal complex in aqueous solution, which influences both the overall charge of the metal complex and its reactivity. For these reasons, we sought to determine the p*K*_a_ values of Os245′ and compare them to those of Ru265′. Spectrophotometric titration of solutions of Os245′ over the range of pH 1–8.5 revealed two p*K*_a_ values of 4.77(01) and 6.41(09), corresponding to sequential deprotonation of the two coordinated water ligands (Fig. S12–S14, ESI†). Notably, the coordinated water molecules of this complex are more acidic than those of its Ru analogue, which is consistent with previous studies investigating the aqueous speciation of Ru and Os arene complexes.^43^ The pH-dependent speciation diagram for Os245′ (Fig. S14, ESI†) shows that the compound exists primarily as the hydroxido-capped species [(HO)(NH_3_)_4_Os(μ-N)Os(NH_3_)_4_(OH)]^3+^ with a small amount of the asymmetric hydroxido-aqua-capped species [(H_2_O)(NH_3_)_4_Os(μ-N)Os(NH_3_)_4_(OH)]^4+^ present in a ratio of approximately 92 : 8. For comparison, the hydroxido and asymmetric hydroxide-aqua-capped species of Ru265′ exist in a ratio of 72 : 28 at pH 7.4. Because glutathione (GSH), a cysteine-containing tripeptide, is present in high concentrations within mammalian cells and has a high affinity for heavy metal ions, we assessed its ability to interact with Os245′. A 4-fold excess of GSH was added to a pH 7.4 phosphate-buffered solution of Os245′, and the resulting solution was probed by ^1^H NMR spectroscopy. After 24 h at 37 °C, no significant spectral changes were observed, indicating that Os245′ does not interact significantly with GSH over this timescale.

**Fig. 3:**
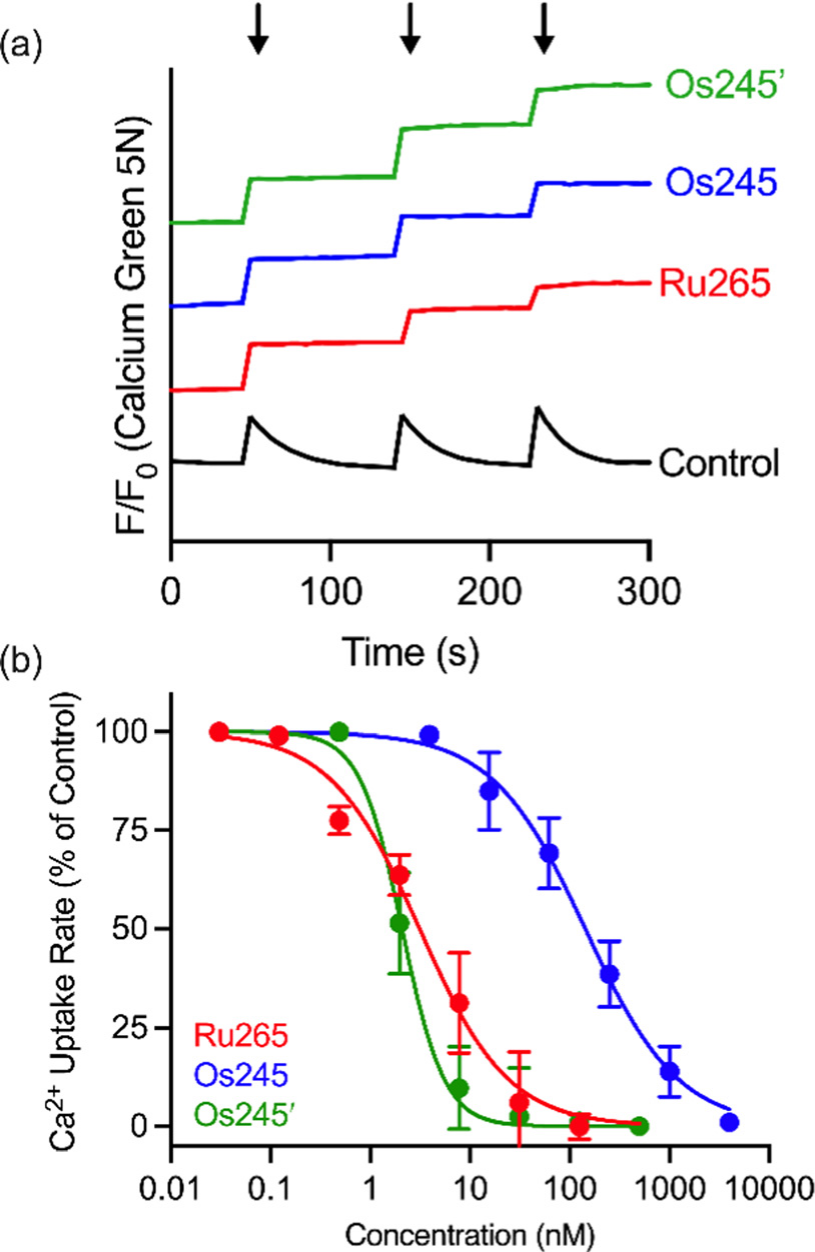
(a) Representative traces of extramitochondrial Ca^2+^ clearance in permeabilized HeLa cells (5 × 10^6^ cells mL^−1^) treated with 1 μM of Ru265, Os245 or Os245′. Arrows indicate addition of 10 μM Ca^2+^. The traces are vertically offset for clarity. (b) Dose-response curve for mt-Ca^2+^ uptake inhibition by Ru265, Os245, and Os245′ in permeabilized HeLa cells. Data are represented as the mean ± SD (*n* = 4–5).

Having fully characterized Os245 and Os245′, we next probed the ability of these compounds to inhibit mt-Ca^2+^ uptake in permeabilized cells. Following previously described protocols, HeLa cells were permeabilized using digitonin and treated with the complex in the presence of the Ca^2+^-responsive fluorescent dye Calcium Green 5N (CGN; 1 μM).^18–21^ In untreated cells, the addition of Ca^2+^ elicits a sharp increase in the fluorescence intensity of CGN, which then follows an exponential decay as the mitochondria sequester Ca^2+^ away from the dye. In the presence of Os245 or Os245′ (1 μM), the initial rise in fluorescence upon Ca^2+^ addition is observed but does not decrease, which indicates that these compounds effectively block MCU-mediated mt-Ca^2+^ uptake (Fig. 3).

We next performed a dose-response analysis to determine the relative potency of Os245 towards MCU inhibition (Fig. 3b). Permeabilized cells were treated with varying concentrations of each complex, which was diluted from a freshly prepared stock solution, and the relative rate of mt-Ca^2+^ accumulation at each concentration was normalized to that of untreated control cells. The IC_50_ for MCU inhibition by Os245 was found to be 103 nM, signifying it to be 100-fold less potent than Ru265 (Table 2).^19^ In contrast, Os245′ is an equally potent MCU inhibitor as Ru265, suggesting that the axial ligands play an important role in the mechanism of MCU inhibition among this class of complexes. This hypothesis was supported by molecular docking calculations, which we performed using the highly conserved DIME region located in cytosolic entrance of the MCU pore as the search space. The choice of search space was justified based on our previous studies which have demonstrated that the known MCU inhibitors Ru265, Ru360, and mitoxantrone all inhibit mt-Ca^2+^ uptake through interactions with this region of the MCU pore.^12,19,44^ The docking calculations predict significant hydrogen-bonding interactions between both the ammine and hydroxido ligands of Os245′ and the DIME-Asp (D261) and -Glu (E264) residues of the MCU pore (Fig. S16, ESI†). In contrast, our simulations indicate that only the ammine, but not chlorido, ligands of Os245 engage in hydrogen-bonding interactions with the DIME region of the MCU, leading to a lower docking score for this complex (Table S6, ESI†). These results suggest that the enhanced potency of Os245′ towards MCU inhibition compared to Os245 may be a consequence of the additional hydrogen-bonding interactions that occur between the axial aqua/hydroxido ligands and the amino acid side chains of the DIME region of the MCU pore.

**Table 2:** Mitochondrial Ca^2+^ uptake inhibition IC_50_ values of Ru265, Os245, and Os245′ in permeabilized HeLa cells (5 × 10^6^ cells mL^−1^). Data are reported as the mean ± SD (*n* = 4–5)

Complex	IC_50_ (nM)
Ru265^a^	3.9 ± 1
Os245	103 ± 30
Os245′	2.3 ± 0.8

a Ref. 19.

**Fig. 4:**
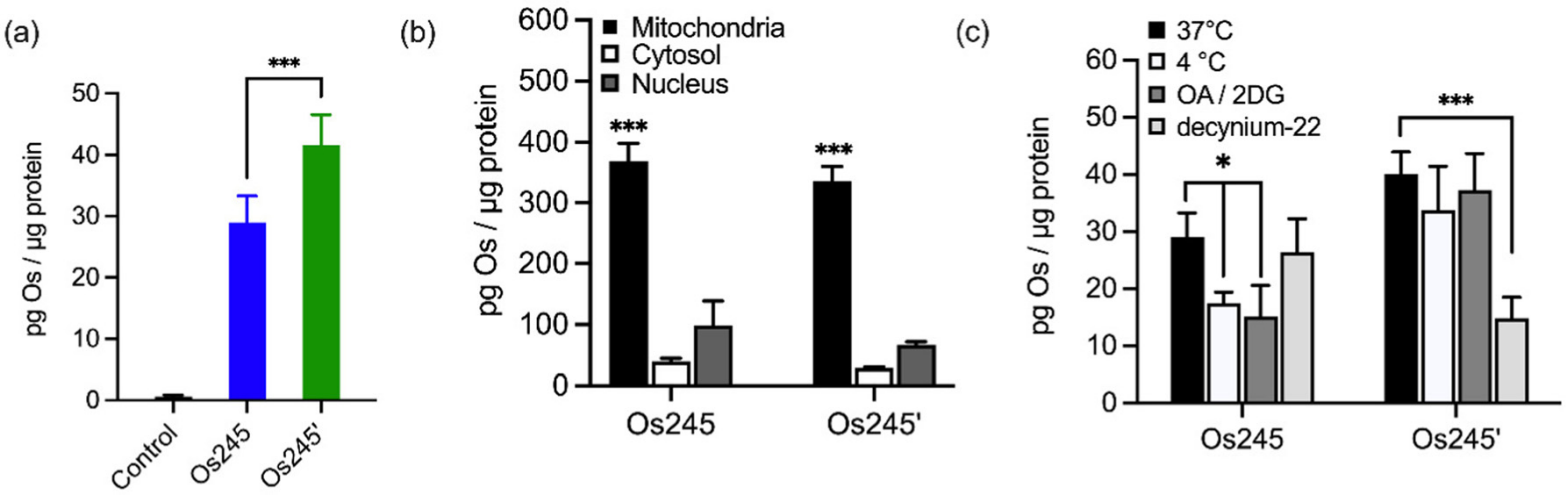
(a) Cellular Os accumulation in HeLa cells treated with Os245 or Os245′ (50 μM, 3 h dose) at 37 °C. (b) Comparison of Os content in the mitochondria, cytosol, and nuclei of HeLa cells treated with Os245 or Os245′ (50 μM, 24 h dose). (c) Cellular uptake of Os245 and Os245′ (50 μM, 3 h dose) under the following incubation conditions: (black) 37 °C; (white) 4 °C; (dark grey) 50 mM 2-deoxy-d-glucose and 5 μM oligomycin A; (light grey) 1 μM decynium-22. Data are represented as the mean ± SD. *p < 0.05, ***p < 0.005, ****p < 0.001 as determined by a two-tailed Student's *t*-test (*n* = 3–5).

Given the sub-micromolar MCU-inhibitory activity of these Os complexes in permeabilized cells, we next sought to investigate their potential use in intact cells. To assess its suitability for this application, we measured the cellular Os accumulation in HeLa cells treated with 50 μM Os245 for 3 h at 37 °C. In contrast to our studies with Ru and Co, which employed graphite furnace atomic absorption spectroscopy (GFAAS),^19–21,45^ these studies required the use of inductively coupled plasma mass spectrometry (ICP-MS) because Os forms non-volatile metallic Os or Os-carbide species within the graphite furnace.^46–48^ Under these conditions, we observed a 30-fold increase of intracellular Os compared to untreated cells, in which the measured quantities of Os were below the detection limit (Fig. 4a). Cells treated with Os245′ contained approximately 2-fold higher Os content compared to cells treated with the chlorido-capped analogue. Additionally, cell fractionation experiments revealed that both Os complexes localize in the mitochondria compared to the cytosol or nucleus (Fig. 4b).

To address the question of why Os245′ is more cell permeable than Os245, we sought to investigate the mechanism of uptake of the compounds. When cells were incubated with the complex at 4 °C or in the presence of 2-deoxy-d-glucose (50 mM) and oligomycin A (5 μM), conditions that diminish energy-dependent cellular uptake pathways, we observed a 2-fold decrease in cellular Os content in Os245-treated cells, but no significant differences for those treated with Os245′ (Fig. 4c). These results suggest that the chlorido-capped complex may cross the cell membrane in an energy-dependent manner, whereas the aqua-capped complex does not. Given that Ru265 enters cells *via* OCT3,^21^ we investigated whether the Os analogues are also substrates for this transporter by measuring their accumulation in HeLa cells in the presence of the OCT3 inhibitor decynium-22.^49,50^ The cellular accumulation of Os245 in the presence of decynium-22 was not significantly diminished in comparison to cells incubated in the absence of the OCT3 inhibitor, suggesting that OCT3 is not involved in its uptake. In contrast, the Os content of cells treated with Os245′ is significantly reduced in the presence of decynium-22, implicating this compound to be a substrate for OCT3. Previous studies have reported a change in the cellular uptake pathways among structurally similar metal complexes with different ligands and the disparate uptake pathways have been attributed to a change in polarization of the complex.^51–54^ Given that the presence of hydrogen-bond acceptors is an important factor in substrate binding to organic cation and anion transporters,^55,56^ it is possible that the terminal aqua/hydroxido ligands of Os245′ make this complex a better substrate for OCT3 than its chlorido-capped analogue. These results highlight how the greater inertness of Os complexes towards ligand substitution compared to their Ru analogues allowed us to study the role of the axial ligands on the biological activity of this class of compounds, a feat that was not previously possible due to the rapid ligand exchange kinetics of Ru265 in aqueous solution.

**Fig. 5:**
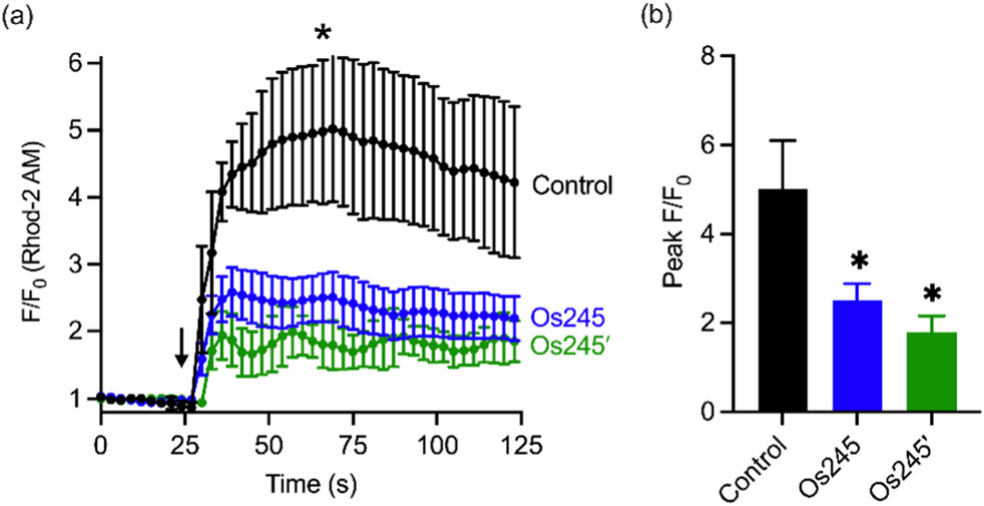
(a) Mitochondrial Ca^2+^ accumulation after addition of 100 μM histamine in HeLa cells pretreated with 0 or 50 μM of Os245 or Os245′ for 1 h and then loaded with 2 μM Rhod2AM. The arrow indicates the time of histamine addition. (b) Quantification of peak *F*/*F*_0_ at the point indicated by the asterisk in panel (a). Data are represented as the mean ± SD. *p < 0.05 as determined by a two-tailed Student's *t*-test (*n* = 2 independent biological replicates).

Considering the MCU-inhibitory properties and good cell permeability of Os245 and Os245′, we hypothesized that these complexes could be used to inhibit the MCU in intact cells. As described in our previous studies with Ru265,^19^ this property was assessed by loading HeLa cells with the mitochondrial-localizing dye Rhod 2-AM^57^ and treating them with either 0 or 50 μM of the complexes for 1 h, prior to simulating mt-Ca^2+^ uptake with histamine (100 μM). Cells treated with either Os245 or Os245′ show decreased mt-Ca^2+^ levels compared to control cells (Fig. 5). Even though Os245 is less potent than Ru265 towards MCU-inhibition in permeabilized cells, this compound retains the ability to inhibit mt-Ca^2+^ uptake in intact cells.

**Fig. 6:**
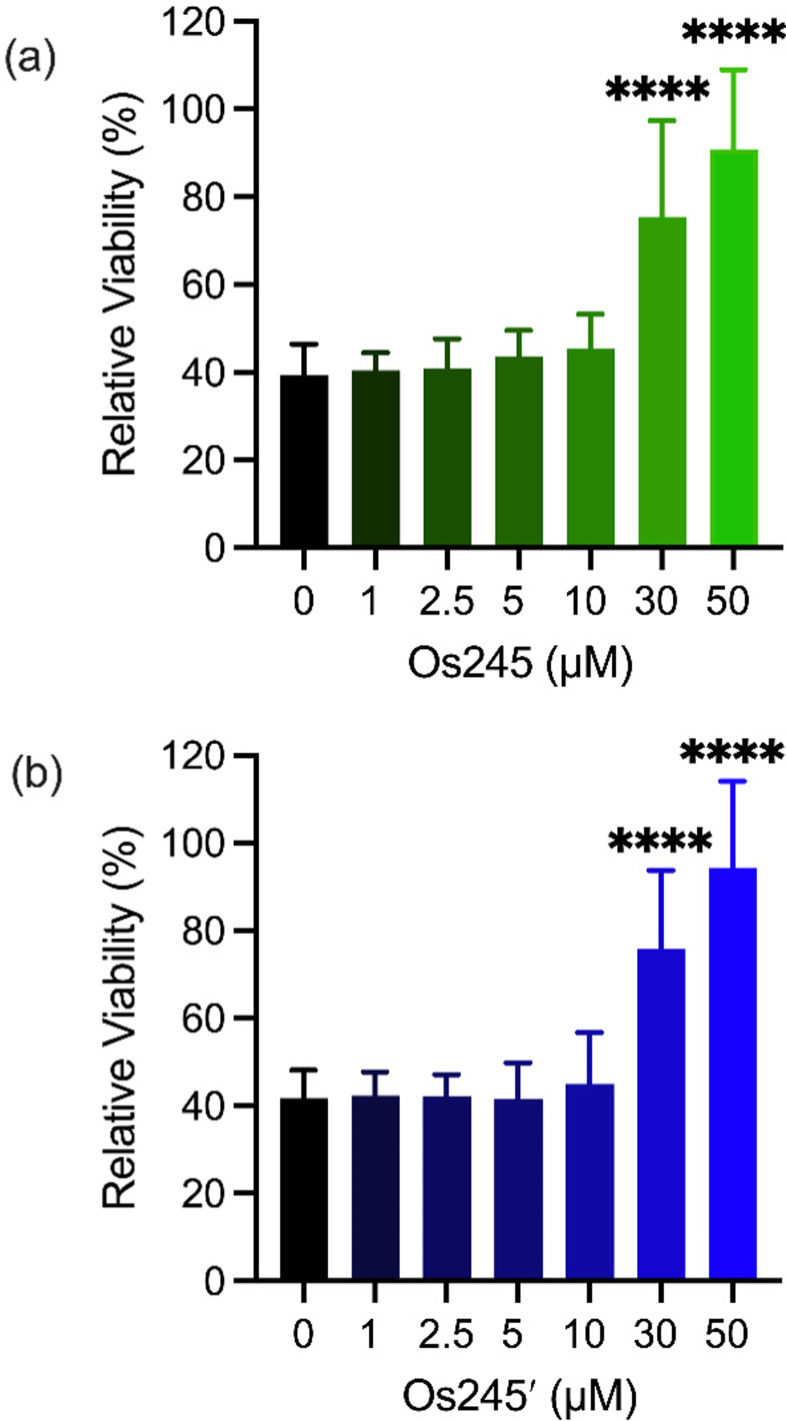
Cell viability in primary cortical neuron cultures treated with increasing concentrations of Os245 or Os245′ for 3 h before being subjected to a lethal period of OGD (90 min). Cell viability was measured using the MTT assay 24 h after OGD. Bars represent the mean + SD. ****p < 0.0001 relative to vehicle (0 μM) as determined by Mann–Whitney *U* tests (*n* = 4).

Metal ions regulate a wide range of processes within neurobiology, and mt-Ca^2+^ overload, in particular, gives rise to neuronal cell death after ischemic stroke.^58^ Encouraged by the cell-permeability of these Os complexes and their ability to inhibit the MCU in intact cells, we hypothesized that, like Ru265, Os245 and Os245′ would protect cortical neurons against the mt-Ca^2+^ overload responsible for viability loss after exposure to a lethal period of OGD, an *in vitro* model of ischemic stroke.^27^ Cellular uptake studies in these cortical neurons, following the procedures described above, revealed that both complexes retain their membrane permeability in these cells. Consistent with our observations in HeLa cells, Os245′ accumulated in these cells to a greater extent than Os245 (Fig. S17 and S18, ESI†). After verifying their cell permeability in neurons, we next investigated the ability of Os245 and Os245′ to protect these cells against OGD. Neurons were treated with varying concentrations of each complex for 3 h prior to exposure to OGD for 90 min. The viability of these cells was then measured 24 h later by the 3-(4,5-dimethylthiazol-2-yl)-2,5-diphenyltetrazolium bromide (MTT) assay. Both compounds elicited a concentration-dependent increase in cell viability up to 50 μM (Fig. 6), indicating that these new MCU inhibitors can protect neurons from the toxic mt-Ca^2+^ overload responsible for viability loss after a lethal period of OGD. Compared to Ru265, however, Os245′ is approximately three-fold less effective at preserving cell viability in the OGD model. This result is unexpected given that these compounds show similar cell uptake and potency at inhibiting the MCU. The origin of their differential cytoprotective effects is uncertain but may be a result of different intracellular target-binding or slower reaction kinetics. Further studies are currently underway to understand the cellular processing of Os245′.

**Fig. 7:**
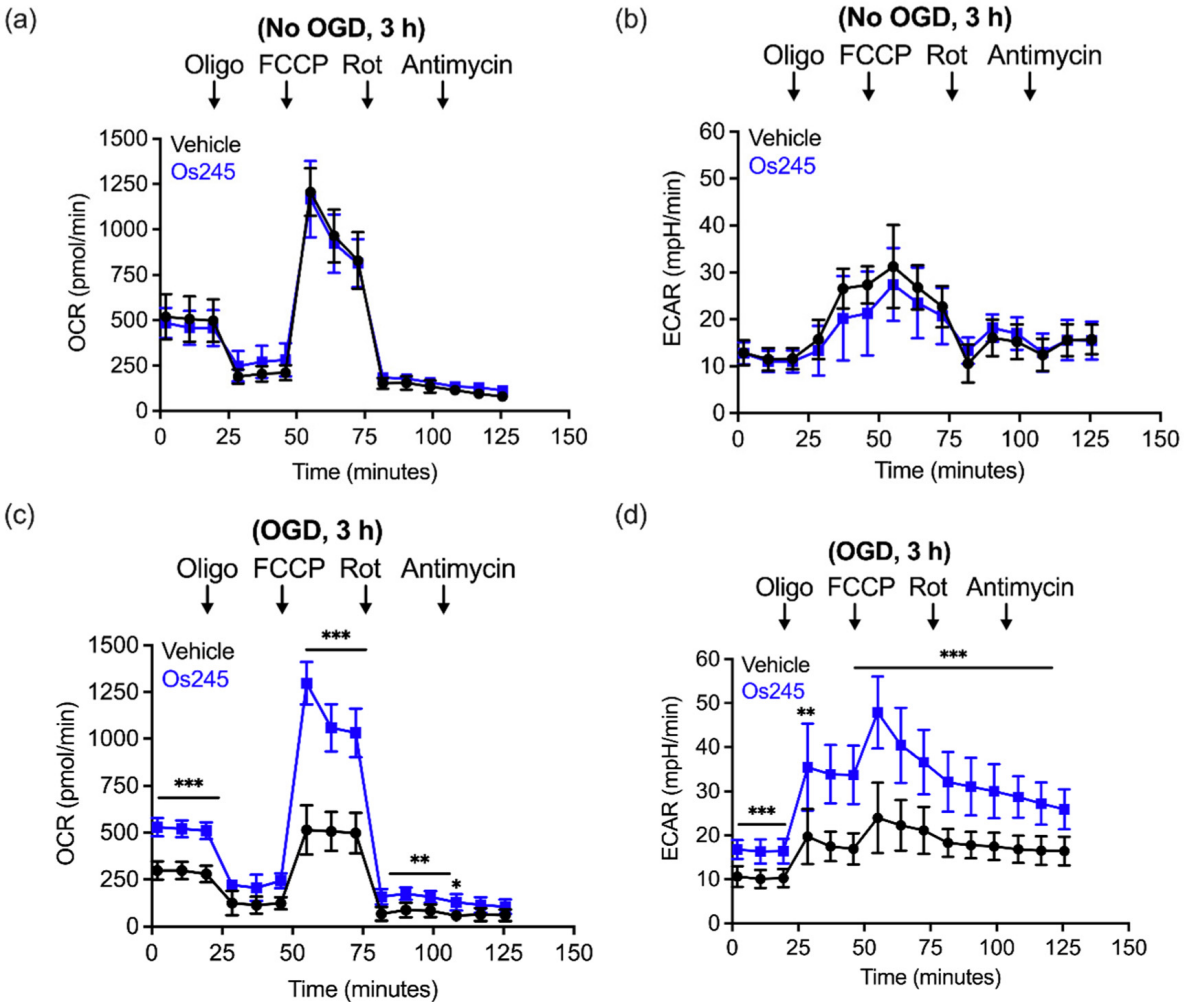
(a) Oxygen consumption rate (OCR) and (b) extracellular acidification rate (ECAR) measurements in primary cortical neuron cultures after the sequential addition of oligomycin (1 μM), FCCP (2 μM), rotenone (300 mM), and antimycin (1 μM) following treatment with Os245 (75 μM) for 3 h. (c and d) Pretreatment with Os245 (75 μM) for 3 h preserves (c) OCR and (d) ECAR in primary cortical neurons 2 h after being subjected to 30 min OGD. Data is represented as the mean ± SD of data. *p < 0.05, **p < 0.01 and ***p < 0.001 as determined by two-way ANOVA followed by Bonferroni's *post hoc* test (*n* = 3).

We further assessed the influence of these compounds on mitochondrial function and glycolysis in neurons subjected to OGD using a Seahorse extracellular flux analyzer.^27^ Treatment of cortical neurons with Os245 or Os245′ (75 μM) for 3 h under normal culture conditions did not alter the basal oxygen consumption rate (OCR), the carbonyl cyanide-4-(trifluoromethoxy) phenylhydrazone (FCCP)-induced maximal OCR, or residual OCR after the addition of rotenone and antimycin. Compared to control neuronal cultures, the extracellular acidification rate (ECAR) of these cells was also unchanged in the presence of Os245 or Os245′ (75 μM), indicating that these complexes did not alter basal glycolysis (Fig. 7 and Fig. S19, ESI†). These findings show that neither Os245 nor Os245′ negatively affect neuronal bioenergetics. Consistent with the suppression of neuronal bioenergetics by an *in vitro* ischemic stroke, OCR and ECAR were significantly reduced 2 h after exposure to 30 min of OGD. In contrast, treatment with 75 μM of Os245 or Os245′ for 3 h prior to OGD preserved mitochondrial respiration and glycolysis (Fig. 7 and Fig. S19, ESI†). Based on the abilities of Os245 and Os245′ to inhibit mt-Ca^2+^ uptake in intact cells, our neuronal bioenergetic measurements suggest that these novel MCU inhibitors preserved mitochondrial function by preventing toxic mt-Ca^2+^ overload.^59,60^

**Fig. 8:**
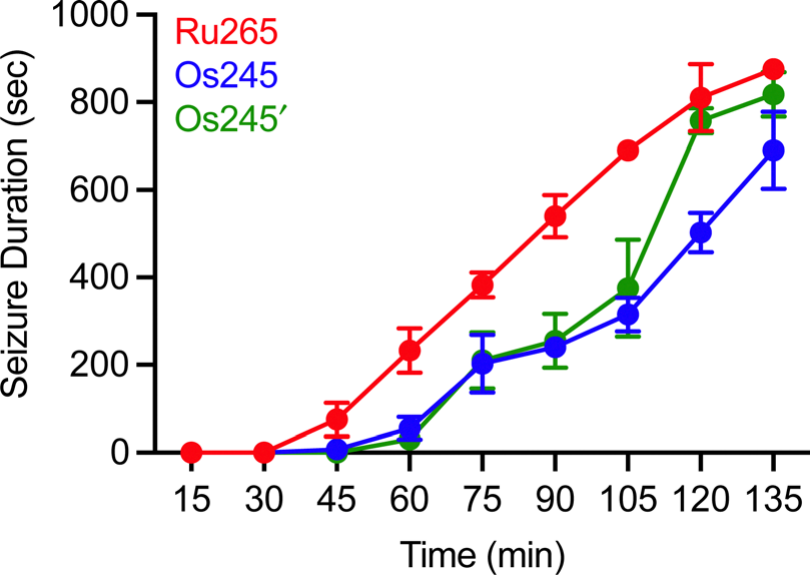
Seizure duration in male C57/BI6 mice injected i.p. with 10 mg kg^−1^ of Ru265, Os245, or Os245′.

Although MCU inhibitors have shown promise as therapeutic candidates for the acute management of ischemic stroke, it has been shown that *in vivo* administration of the commonly used MCU inhibitor ruthenium red induces seizures in rats.^61,62^ Similarly, we have reported that Ru265 induces seizure-like behaviors in mice when injected intraperitoneally (i.p.) at a dose of 10 mg kg^−1^ and lethal convulsions at dose of 30 mg kg^−1^ (i.p.).^27^ The mechanism by which these compounds promote proconvulsant activities is currently unknown, but could be a consequence of MCU-inhibition^27,61–63^ or the result of off-target activities. Because Os245 is a less potent MCU inhibitor and has slower reaction kinetics compared to Ru265, we speculated that it may be less likely to cause seizures *in vivo*. To evaluate this hypothesis, male C57/BI6 mice were injected with 10 mg kg^−1^ (i.p.) of either Ru265, Os245, or Os245′, and then monitored for seizure-like behaviors (whisker trembling, motionless starting, facial jerking, and clonic convulsions). In line with our previous study, mice injected with Ru265 at a dose of 10 mg kg^−1^ (i.p.) displayed seizure-like behaviors approximately 45 min after injection (Fig. 8). In the case of Os245 and Os245′, the onset of these seizure-like behaviors was significantly delayed compared to Ru265. For example, seizures lasting 400 s were observed in mice 75 min post-injection of Ru265, whereas it took 30 min longer for the Os-treated mice to display seizures of the same duration (Fig. 8). The severity of these seizures increased over time and all animals had to be euthanized approximately 3 h after injection. This delayed seizure response upon administration of the Os complexes compared to Ru265 may likely be a consequence of their slower ligand substitution rates. Importantly, the slower seizure onset may have significant implications in the therapeutic use of these compounds.

## Conclusions

The nitrido-bridged ruthenium complex Ru265 effectively inhibits MCU-mediated mt-Ca^2+^ uptake both *in vitro* and *in vivo*.^19–21,27^ In this report, we have shown that the isostructural Os-based analogues of Ru265 and Ru265′, Os245 and Os245′, are also potent and cell-permeable inhibitors of the MCU. We provide an improved synthetic procedure for the preparation of analytically pure Os245 and Os245′ and have fully characterized the complexes in direct comparison to Ru265. Although the Ru and Os complexes are comparable in most of their properties, a notable difference arises within their ligand substitution kinetics. As expected, based on the known differences between 4d and 5d transition metal complexes, the aquation of Os245 is two orders of magnitude slower than that of Ru265. The slow axial ligand exchange kinetics allowed us to compare the biological activity of the chlorido and aqua-capped species *in vitro* and *in vivo*, revealing the latter complex to be an approximately 100-fold more potent MCU inhibitor. Docking calculations of both complexes with the DIME region of the MCU also support this observation, as the axial aqua/hydroxido ligands of Os245′ engage in effective hydrogen-bonding interactions with the glutamate amino acid residues within the cytosolic pore of the MCU channel. Cell-based studies using both HeLa cells and cortical neurons demonstrate that these Os complexes are cell permeable and inhibit the MCU in intact cells. These properties were further leveraged to show Os245 and Os245′ preserved mitochondrial respiration and glycolysis of neurons in the OGD model of toxic mt-Ca^2+^ overloading. These results highlight the therapeutic potential of MCU inhibition for the prevention of ischemic reperfusion injury.^6,64–66^ The cytoprotective effects of these Os complexes in primary cultures of mouse cortical neurons are noteworthy given the acute toxicity of OsO_4_ and numerous osmium coordination compounds.^67–70^ As with Ru265, this low toxicity demonstrates how the coordination geometry, oxidation state, and molecular structure of metal-based compounds greatly influences their biological activities.^20,21^

Like ruthenium red and Ru265,^27,61,62^ Os245 and Os245′ induced seizure-like behaviors in mice. Initially, it was not immediately apparent if this adverse side effect is mediated by MCU inhibition^63^ or the result of binding to an off-target biomolecule. More recent mechanistic studies suggest that the observed proconvulsant activity of Ru265 is due to off-target binding of this complex.^71^ The observation that seizure onset is delayed in mice treated with Os245 and Os245′ suggests that this adverse side effect can be mitigated with the appropriate compound design strategies. Work is currently underway in our labs to identify the mechanism by which this class of compounds induces seizures. Once the source of the proconvulsant activity of this class of compounds has been identified, it should be possible to develop a counter screen to identify MCU inhibitors with an improved therapeutic index. Furthermore, optimizing the delivery of Os245 and Os245′ to ischemic regions of the brain may be an effective means to further minimize seizure activity.

In summary, this work highlights how the identity of the axial ligands can dictate the biological activity of structurally similar complexes. This phenomenon could not be studied with Ru265 owing to its fast aquation of the axial chlorido ligands in biological solutions. The greater inertness of Os towards ligand substitution was utilized to address this question and design new MCU inhibitors with different axial ligands. Knowledge of how the structure and physical properties of these complexes relate to their mechanism of cellular uptake and MCU inhibition will be applied in the design of future inhibitors and drug delivery strategies for a variety of therapeutic applications. For example, we have recently studied how Ru265 analogues bearing axial carboxylate ligands can act as prodrugs for this compound.^72^

## Conflicts of interest

The authors have no conflict of interest to declare.

## Supplementary Material

CB-004-D2CB00189F-s001

CB-004-D2CB00189F-s002
