# Supplementary material for: Dinuclear nitrido-bridged osmium complexes inhibit the mitochondrial calcium uniporter and protect cortical neurons against lethal oxygen–glucose deprivation
Source: RSC Chem Biol. 2022 Nov 14;4(1):84–93. doi: 10.1039/d2cb00189f (PMC9811523; doi:10.1039/d2cb00189f)
Supplement: CB-004-D2CB00189F-s001 [file CB-004-D2CB00189F-s001.pdf]

Electronic Supplementary Material (ESI) for RSC Chemical Biology.
This journal is © The Royal Society of Chemistry 2022

Supporting Information for:

Dinuclear Nitrido-Bridged Osmium Complexes Inhibit the Mitochondrial Calcium Uniporter and Protect Cortical Neurons Against Lethal Oxygen Glucose Deprivation.

Joshua J. Woods,[1,2,†,‡] Robyn J. Novorolsky,[3,4,†] Nicholas P. Bigham,[1] George S. Robertson,[3,4,5] and Justin J. Wilson[1,*]

[1]Department of Chemistry and Chemical Biology, Cornell University, Ithaca, NY, 14853. USA.

[2]Robert F. Smith School for Chemical and Biomolecular Engineering, Cornell University, Ithaca, NY, 14853. USA.

[3]Department of Pharmacology, Faculty of Medicine, Dalhousie University, Life Sciences Research Institute, Halifax, NS B3H 0A8. Canada.

[4]Brain Repair Centre, Faculty of Medicine, Dalhousie University, Life Sciences Research Institute. Halifax, NS B3H 0A8. Canada.

[5]Department of Psychiatry, Faculty of Medicine, Dalhousie University, Life Sciences Research Institute, Halifax, NS B3H0A8. Canada.

[†]Equal Contribution

[‡]Present Address: Chemical Sciences Division, Lawrence Berkeley National Laboratory, Berkeley, CA 94720

*Corresponding Authors: J. J. Wilson: jjw275@cornell.edu; G. S. Robertson: George.Robertson@Dal.Ca

S1. GENERAL CONSIDERATIONS

All reagents and solvents were obtained commercially and used without further purification unless otherwise noted. Deionized water (≥18 MΩ·cm) was obtained from an Elga Purelab Flex 2 water purification system.

NMR spectra were obtained at 25 °C using a Bruker AV III HD 500 MHz spectrometer equipped with a broadband Prodigy cryoprobe. Chemical shifts are reported in ppm and were referenced to the residual solvent signal vs tetramethylsilane (TMS) at 0 ppm. The ^{19}F spectra were referenced indirectly using the absolute reference function provided in MestReNova (MestraLab Research). UV-vis spectra were recorded using 1 cm quartz cuvettes and a Shimadzu UV-1900 spectrometer (Shimadzu, Kyoto, Japan) fitted with a temperature-controlled circulating water bath. Infrared spectra were recorded using a Bruker Tensor II Infrared Spectrometer equipped with a diamond ATR crystal (Bruker, Billerica, MA). Elemental analyses (C, H, N) were carried out by Atlantic Microlab Inc. (Norcross, GA). Cyclic voltammetry measurements were carried out using a Pine WaveNow potentiostat with a three-electrode setup consisting of a glassy carbon working electrode, a platinum counter electrode, and a Ag/AgCl quasi-reference electrode (Pine Instruments). The complexes were dissolved water containing 0.1 M KCl and 10 mM HEPES (pH 7.4). All potentials were referenced to the $[Ru(NH_3)_6]^{3+/2+}$ redox couple at -0.193 V vs the saturated calomel electrode (SCE) as an internal standard.[1] The sample cell was deoxygenated by bubbling nitrogen gas through the solution for 15 min prior to analysis and maintained under a blanket of nitrogen during the experiment.

Inductively coupled mass spectrometry (ICP-MS) was performed using an Agilent ICP-MS 7800 fitted with a Micromist nebulizer with a sample uptake rate of 0.4 mL min^{-1}. Ni was used as the cone material. The Os content was determined by tracking ^{189}Os and a series of standardized solutions containing 0–100 µg L^{-1} Os, which were prepared from a standardized ICP-MS solution of Os (1000 ± 5 µg mL^{-1} in 20% HCl (Agilent Technologies, Santa Clara, CA). All calibration standards and samples were prepared in a stabilizing solution containing 500 µM each of ethylenediaminetetraacetic acid (EDTA), thiourea, and ascorbic acid to prevent the loss of Os as volatile OsO_4 under the oxidizing conditions required for ICP-MS.[2]

All studies involving animals were conducted in agreement with the guidelines set by the Canadian Council on Animal Care (CCAC) and approved by the University Committee on Laboratory Animals (UCLA) at Dalhousie University.

S2. SYNTHESIS AND CHARACTERIZATION

Caution! Os complexes can be converted to toxic OsO_4 under strongly oxidizing conditions. Avoid contact with oxidizing agents.

$[Os_2(\mu\text{-}N)(NH_3)_8Cl_2]Cl_3$ (Os245). In a typical procedure, 0.500 g (1.14 mmol) of $(NH_4)_2OsCl_6$ was placed in a thick-walled pressure tube (35 mL capacity) with 5 mL of concentrated NH_4OH. The reaction vessel was sealed and heated behind a blast shield for 72 h. The reaction mixture was allowed to cool and filtered to remove a dark brown precipitate. The dark yellow filtrate was concentrated to approximately 2.5 mL, and 4 mL of concentrated HCl was added to precipitate microcrystalline yellow powder (~150 mg). After cooling to 0 °C, the precipitate was collected by filtration and suspended in 10 mL of 4M HCl and heated at reflux for 12 h. The resulting precipitate was collected by filtration, washed with 2 × 10 mL EtOH, and dried under vacuum. The amount of **1** and **2** remaining in the product was monitored by NMR spectroscopy. In most cases, full conversion was complete after 48 h refluxing in 4 M HCl. Yield: 0.115 g (0.162 mmol, 28.4%). ^{1}H NMR (500 MHz, DMSO-d_6, 25 °C) δ = 5.03 (s, 24H) ppm. IR (ATR) ν = 3230 (m), 3149 (m), 1584 (m), 1320 (s, sh), 1108 (s, sh), 841 (m), 554 (w) cm^{-1}. UV-vis (50 mM MOPS pH 7.4): 245 nm (ε = 50,000 ± 2600 M^{-1} cm^{-1}), 276 nm (ε = 7700 ± 360 M^{-1} cm^{-1}). Elemental Analysis Calcd for $H_{24}Cl_5N_9Os_2\cdot 3H_2O$: C 0; H 3.97; N 16.54. Found C 0.37; H 3.96; N 16.43.

[Os_2(μ-N)$(NH_3)_8(OH_2)_2$]$(OTf)_5$ (Os245′) The complex Os245 (44 mg, 0.063 mmol) was suspended in 10 mL H_2O. A solution of AgOTf (81 mg, 0.32 mmol, 5 equiv.) in 5 mL H_2O was added to the suspension and the reaction mixture was stirred at 50 °C for 48 h while being protected from light. The white precipitate was removed by filtration to give a bright yellow solution that was evaporated to dryness to give a yellow sold that was washed with minimal ice cold EtOH and air dried. Yield: 66 mg (0.05 mmol, 79.3%). ^{1}H NMR (500 MHz, DMSO-d_6, 25 °C) δ = 4.81 (s, 24H, NH_3) ppm. ^{19}F NMR (470 MHz, DMSO-d_6, 25 °C) δ = –78.28 ppm. IR (ATR) ν = 3285 (w, br), 3200 (w, br), 1591 (w, br), 1351 (m), 1218 (s), 1161 (s), 1124 (s), 1023 (s), 824 (m, br), 761 (w), 628 (w), 574 (w) cm^{-1}. UV-vis (50 mM MOPS pH 7.4, ε): 240 nm (43,000 ± 1200 M^{-1} cm^{-1}). Elemental Analysis Calcd for $Os_2S_5O_{17}F_{15}N_9C_5H_{28}$·2EtOH: C 7.70, H 2.87, N 8.98. Found: C 7.67, H 2.56, N 9.00.

S3. SINGLE-CRYSTAL X-RAY DIFFRACTION

Low temperature (100 K) X-ray diffraction data was collected using a Rigaku XtaLAB Synergy diffractometer equipped with a 4-circle Kappa goniometer and HyPix 6000HE Hybrid Photon Counting (HPC) detector with monochromated Mo Kα radiation (λ = 0.71073 Å). Diffraction images were processed using the CrysAlisPro software (Rigaku Oxford Diffraction). The structure was solved through intrinsic phasing using SHELXT[3] and refined against F^2 on all data by full-matrix least-squares with SHELXL[4] following established strategies.[5] All non-hydrogen atoms were refined anisotropically. Hydrogen atoms bound to the ammine ligands in all three structures were included in the model at geometrically calculated positions and refined using a riding model with the isotropic displacement parameters of all hydrogen atoms fixed at 1.2 times the U value of the atoms they are linked to (HFIX 137).

X-ray diffraction quality crystals of Os245 were grown by vapor diffusion of acetone into a solution of the complex dissolved in a saturated aqueous solution of $NaBPh_4$. For Os245, visual analysis of the reflections from the data frames showed no obvious signs of non-merohedral twinning. The initial solution suggested that the crystal belonged to the highly symmetric orthorhombic space group *Cmmm*. Searches for merohedral twinning and pseudosymmetry were unsuccessful and did not yield meaningful results. Upon solving the structure using the intrinsic phasing method of SHELXT, the location of the cation and two of the outer sphere chloride anions (Cl2 and Cl3) were unequivocally established. The location of the third chloride counterion (Cl4) was determined upon refinement of the structure with SHELXL. This atom was found to be disordered over two special positions with symmetry 2/m and *m*. The multiplicity of these two special positions requires that the atom be refined with an occupancy of 1/8 in the unit cell. Attempts to mitigate the disorder using the PART -1 command yielded nonsensical solutions and this atom was refined at 1/8 occupancy in the unit cell. Another Q peak corresponding to a solvent water molecule was found in the density difference map after assignment of Cl4. This atom was found to be similarly disordered over two special positions of symmetry *m* and *mm* and was refined with 1/8 occupancy. Due to this disorder, the hydrogen atoms of the water molecule could not be accurately located in the density difference map and were not included in the model. One residual Q peak corresponding to 2.6 e^- is located near the central nitrogen atom of the Os–N–Os moiety and has been left unrefined, generating one B-level alert. Peaks with higher residual density are often observed in the presence of heavy atoms due to Fourier truncation.

Crystals of **1** were grown by vapor diffusion of tetrahydrofuran into a dilute methanesulfonic acid solution of the crude product obtained from the reaction between $(NH_4)_2OsCl_6$ and NH_4OH (Table S1, Entry 2). In the structure of **1**, the hydrogen atoms of the solvent water molecule were located in the electron density difference map and refined semi-freely constraining the O–H distance to be 0.84 Å and the thermal parameters to be 1.2 times that of the oxygen atom that they are bound to.

The triflate salt of Os245′ was converted to the nitrate salt by careful dropwise addition of dilute (~0.1 M) HNO_3 to a concentrated aqueous solution of the complex at 0 °C. The yellow microcrystalline precipitate was collected by centrifugation and redissolved in a minimal volume of water. Diffraction quality crystals of this complex were obtained by vapor diffusion of acetone into this aqueous solution. In the structure of Os245′, the hydrogen atoms of the solvent water molecule were located in the electron density difference map and refined semi-freely constraining the O–H distance to be 0.84 Å and the thermal parameters to be 1.2 times that of the oxygen atom that they are bound to.

S4. AQUATION KINETICS AND SOLUTION THERMODYNAMICS

Aquation Kinetics

A stock solution of Os245 in pure H_2O (1.5 mM) was diluted to a final concentration of 25 µM in 50 mM MOPS (pH 7.4) supplemented with 0 or 0.15 M NaCl in a quartz cuvette, which was placed in a water-jacketed cuvette holder and incubated at 37 °C. The absorbance spectrum of the solution was recorded every 1 h over a period of 24 h. The pseudo-first order overall rate constant k_{obs} (s^{-1}) was determined by fitting the decrease in absorbance at 245 nm as a single exponential decay.

Interaction of Glutathione (GSH) with Os245

A solution of Os245 (2.5 mg) was prepared in 1 mL 90% pH 7.4 phosphate-buffered saline (PBS, Corning Life Sciences), 10% D_2O, and < 1% dioxane as an internal reference. A 4-fold excess of GSH (4.3 mg) was dissolved into the solution, and 600 µL of the solution was transferred to an NMR tube. The solution was then heated at 37 °C for 24 h before water suppression ^1H NMR (500 MHz, 25 °C) was taken. For comparison, the same concentration GSH without Os245 was prepared under the same conditions.

Spectrophotometric Titration

Spectrophotometric titration of Os245′ was carried out following previously published procedures[6] using a Metrohm Titrando 888 titrator equipped with a Ross Orion combination electrode (8103BN, Thermofisher Scientific), a Metrohm 806 exchange unit with an automatic burette and *Tiamo 2.5* software. The titration vessel was fitted with a removable glass insert (~70 mL) and was held at 25 °C using a circulating water bath. CO_2 was excluded from the vessel using a small positive pressure of argon scrubbed with 30 wt % KOH. Carbonate-free KOH (~0.1 M) was prepared by the dissolution of pellets (semiconductor grade, 99.99% trace metals basis, Sigma-Aldrich) in freshly boiled water and standardized by potentiometric titration against potassium hydrogen phthalate. HCl (0.1 M) was obtained as a standardized solution (VWR Chemicals BDH) and standardized by potentiometric titration against TRIS base. Before each titration, the electrode was calibrated in terms of the hydrogen-ion concentration by titrating a solution of standardized HCl (5 mM; I = 0.1 M KCl) with standardized KOH. Data within the pH ranges of 2.3−3.2 and 10.8−11.2 were analyzed using the program *Glee* (version 3.0.21)[7] to obtain the standard electrode potential (E_0) and slope factor. The H_2O ion product (pK_w = 13.78) was taken from literature.

For the spectrophotometric titration of Os245′**,** stock solutions of the complex were prepared in pure water and were diluted to a final concentration of 25 µM in water containing 500 µM (20 equiv.) each of TRIS and HEPES. The solution was then treated with 10 mM HCl and the ionic strength of the solution was adjusted to 0.1 M with KCl. Following addition of 0.01–1 mL aliquots of KOH, the solution was allowed to equilibrate for 200 s before the pH was recorded and an aliquot (2 mL) of the titration solution was removed and analyzed by UV-vis. The spectroscopic titration data was analyzed using the program HypSpec2014 over the wavelength of 220–300 nm. The protonation constants were calculated from the average of three independent titrations using

independently prepared stock solutions from at least two synthetic preparations of complex. Control experiments showed no evidence for chloride binding when the aqua complex was incubated in 0.1 M KCl for the duration of the experiment (~3 h) or after 24 h. Attempts to perform the titrations in 0.1 M KNO_3 were unsuccessful as the charge transfer bands of the Os complexes were obstructed by absorbance of NO_3^- in the same region of the UV-vis spectra.

S5. MITOCHONDRIAL CALCIUM UPTAKE STUDIES

Mitochondrial Ca^{2+} Uptake Studies in Permeabilized HeLa Cells.

HeLa cells were grown to near confluency in a 10 cm^2 dish and harvested using 0.05% trypsin + 0.53 mM EDTA (Corning Life Sciences). The cells were pelleted by centrifugation and suspended in cold PBS supplemented with 5 mM EDTA (pH 7.4) and counted using trypan blue. The remaining cells were pelleted by centrifugation at 800 × *g* for 5 min and resuspended in ice cold high KCl solution (125 mM KCl, 20 mM HEPES, 2 mM K_2HPO_4, 5 mM glutamate, 5 mM malate, 1 mM $MgCl_2$, pH 7.2 with KOH) supplemented with 80 µM digitonin and 1 µM thapsigargin. The final solution contained <0.1% DMSO, originating from the digitonin and thapsigargin stocks. The cells were incubated on ice for 15 min and centrifuged at 200 × *g* for 10 min at 4 °C. The pelleted cells were then resuspended in high KCl solution containing 1 µM Calcium Green 5N (ThermoFisher) and 2 mM succinate to a final density of 5 × 10^6 cells/mL. For each experiment, 100 µL of the cell suspension was placed in each well of a black-walled 96 well plate, treated with the desired concentration of the test complex, and allowed to equilibrate at room temperature for ~200 s. The background fluorescence of each well was recorded for 60 s prior to addition of 10 µM $CaCl_2$. The change in fluorescence of the dye (ex: 488; em: 528) in response to Ca^{2+} was recorded every 5 s for at least 120 s or until the fluorescence returned to the baseline. The mitochondrial Ca^{2+} uptake rate was calculated as the slope of the linear fit of the first 25 s of the fluorescence response. Control cells that were not treated with compound were handled identically to the treated cells to account for different incubation lengths. The Ca^{2+} uptake rate of cells was determined by fitting the decay of the Calcium Green 5N signal with a monoexponential decay to obtain the decay constant (k, s^{-1}) and the rate of Ca^{2+} uptake in treated cells was normalized to that of the controls cells. Each replicate was performed using independently prepared cells suspensions to account for differences in cell count. A bicinchoninic acid (BCA) assay was performed at the end of each experiment to confirm similar cell count using the BCA assay kit (ThermoFisher) following manufacturer's instructions. The average protein content of the cell suspensions was 1845 ± 200 µg mL^{-1}. Each replicate was performed using an independently prepared batch of cells and stock solution of complex from at least two independently synthesized batches of compound to account for potential differences in cell count and synthetic preparations.

Mitochondrial Ca^{2+} Uptake in Intact HeLa Cells using Rhod 2-AM.

HeLa cells were seeded in an 8-well chamber µ-slide (Ibidi USA Inc.) at a density of 5 × 10^4 cells mL^{-1} (300 µL $well^{-1}$) and incubated overnight at 37 °C. The following day, cells were treated with the desired metal complex (50 µM) in DMEM supplemented with 10% FBS for 1 h at 37 °C. The culture media was removed, and the cells were washed with 1 × 0.3 mL PBS before the cells were incubated in extracellular medium (ECM; 135 mM NaCl, 20 mM HEPES, 5 mM KCl, 1 mM $MgCl_2$, 1 mM $CaCl_2$) supplemented with 10 mM glucose, 3.2 mg/mL bovine serum albumin (BSA), 0.003% pluronic F127, and 2 µM Rhod 2-AM (Molecular Probes) in the dark for 30 min at room temperature. The ECM was then removed, the cells were washed with 1 × 0.3 mL ECM, and the cells were treated with fresh ECM supplemented with 10 mM glucose and 3.2 mg/mL BSA and incubated for a further 30 min in the dark at room temperature. The buffer was then removed, and the cells washed with 1 × 0.3 mL ECM and treated with ECM supplemented with 10 mM glucose and 3.2 mg/mL BSA at 37 °C for 15 min before imaging using a Zeiss LSM 710 confocal fluorescence microscope using a 40× water objective with an excitation of 561 nm and

an emission window of 568 – 712 nm. After ~ 30 s of baseline recording, histamine (100 µM) was added to the dish and fluorescence images were recorded every 3 s to monitor mt-Ca^{2+} uptake. Images were analyzed and quantified using ImageJ (NIH) and the corrected total cellular fluorescence (CTCF) was calculated using the following formula:

$$CTCF = \text{Integrated density} - (\text{area of cell} \times \text{mean fluorescence of background reading})$$

The average of at least six individual cells was used to determine the average CTCF for reach replicate (4 wells per replicate).

Docking Studies

Docking studies were carried out using the program GOLD[8,9] as previously described.[10] The X-ray crystal structures of Os245 and Os245′ were flexibly docked into the tetrameric human MCU (PDB 6O5B) using all residues within 10 Å of the center of the four aspartate residues of the DIME region of the MCU pore as the search space. Default values for all other parameters were used and the complexes were subjected to 100 genetic algorithm steps using the ChemPLP scoring function to evaluate the results.[8]

S6. CELLULAR ACCUMULATION ASSAYS

Whole Cell Uptake in HeLa Cells

HeLa cells were grown to near confluence in 6-well plates. On the day of the experiment, the culture media was removed, and the cells were treated with fresh media containing 0 or 50 µM complex and incubated for 3 h at 37 °C. The culture media was then removed, and the adherent cells were washed with 3 × 1 mL of PBS and harvested with trypsin + 0.53 mM EDTA before being pelleted by centrifugation (800 × *g* for 10 min). The cell pellet was suspended in 300 µL of ice-cold radioimmunoprecipitation assay (RIPA) lysis buffer (150 mM NaCl, 1% v/v Triton X-100, 0.1% SDS, and 50 mM TRIS, pH 8.0) and incubated for 2 h on ice. The cell lysate was then filtered through a 0.2 µm polyvinylidene difluoride (PVDF) filter to remove particulates and then 200 µL aliquots of the filtrate was immediately diluted to a total volume of 5 mL with stabilization solution (500 µM each of EDTA, thiourea, and ascorbic acid).[2] Samples were kept at room temperature until analysis and were analyzed for Os content by ICP-MS within 7 days of preparation. The protein content of the lysates was determined immediately after filtration using the BCA assay kit. The total Os content of the lysate was normalized by protein content of the sample. Results are reported as the mean mass ratio of total Os to protein content (pg/µg) in each sample ± standard deviation (SD, $n = 3 - 4$).

Whole Cell Uptake in Primary Cortical Neurons

Primary cortical neuron cultures were derived from E16 embryos from time-pregnant CD-1 mice according to our previously described methods.[11] Cortical neuron cultures were seeded at a density of 200,000 cells/well in 24-well plates for uptake studies. After 12 days in culture, neurons were treated with Os245 (10, 30, or 50 µM) or Os245' (10, 30, or 50 µM) for 30 min, 3 h, or 24 h. The media was then removed, and cells were washed with 3 × 500 µL of cold PBS before the addition of 75 µL of RIPA lysis buffer to each well. Plates were then placed on an orbital shaker for 30 min and then cell lysates were collected and centrifuged at 13,000 rpm for 15 min. The protein concentration of the cell lysates was determined immediately using the BCA assay and the remainder of the samples were stored at –80°C for no longer than one week before the Os content was measured using ICP-MS.

Mitochondria and Cytosol Isolation Protocol

Approximately 1 × 10^6 HeLa cells were seeded in two 10 cm^2 culture dishes and allowed to adhere overnight. The following day, the cells were treated with 50 μM complex in normal culture media for 24 h at 37 °C. Following treatment, the cells were washed with 3 × 3 mL PBS and harvested with trypsin + 0.53 mM EDTA. The cells were collected by centrifugation (800 × *g* for 10 min) and suspended in 1 mL of ice cold mitochondrial isolation buffer (pH 7.4) containing 200 mM mannitol, 68 mM sucrose, 50 mM piperazine-*N*,*N′*-bis(2-ethanesulfonic acid), 50 mM KCl, 5 mM EGTA, 2 mM $MgCl_2$, 1 mM dithiothreitol, and 1:500 v/v protease inhibitor cocktail. The cell suspension was then incubated on ice for 20 min before it was homogenized by 40 passes through a 25-guage needle using a 1 mL syringe. The homogenized suspension was centrifuged at 150 × *g* for 10 min. The supernatant was transferred to a clean tube and centrifuged for 10 min at 14,000 × *g* to pellet the mitochondrial fraction. The supernatant of the final centrifugation step was reserved as the cytosolic fraction. The mitochondrial pellet was suspended in 300 μL of RIPA lysis buffer and vortexed at the highest setting for 30 s. The Os content of the mitochondrial lysate and cytosolic fraction was determined by diluting 200 μL of the isolated lysate to a total of 5 mL with stabilization solution, which was then filtered through a 0.2 μm PVDF filter to remove particulates and analyzed by ICP-MS. The Os content of each sample was normalized to the protein content of the sample, which was determined using the BCA assay as described above. Each replicate consisted of lysate solutions combined from two 10 cm^2 culture dishes.

Nucleus Isolation Protocol

Approximately 1 × 10^6 HeLa cells were seeded in two 10 cm^2 culture dishes and allowed to adhere overnight. The following day, the cells were treated with 50 μM complex in normal culture media for 24 h at 37 °C. Following treatment, the cells were washed with 3 × 3 mL PBS and harvested with trypsin + 0.53 mM EDTA. The cells were collected by centrifugation (800 × *g* for 10 min) and suspended in 1 mL of cold hypotonic buffer (pH 7.4) containing 20 mM TRIS·HCl, 10 mM NaCl, and 3 mM $MgCl_2$. The cell suspension was then incubated on ice for 20 min before 50 μL of 10 % v/v NP-40 was added and the solution was vortexed on the highest setting for 15 s. The suspension was centrifuged at 3000 × *g* for 10 min. The pellet was suspended in 500 μL of RIPA lysis buffer, vortexed at the highest setting for 30 s, and incubated on ice for 1 h. The Os content of the nuclear lysate was determined by diluting 200 μL of the isolated lysate to a total of 5 mL with stabilization solution, which was then filtered through a 0.2 μm PVDF filter to remove particulates and analyzed by ICP-MS. The Os content of each sample was normalized to the protein content of the sample, which was determined using the BCA assay as described above. Each replicate consisted of lysate solutions combined from two 10 cm^2 culture dishes.

Role of Temperature in the Cellular Accumulation

HeLa cells were grown to near confluence in 6-well plates. On the day of the experiment, the culture media was removed, and the cells were treated with fresh media containing 0 or 50 μM complex and incubated for 3 h at 37 °C or 4 °C. The culture media was then removed, and the adherent cells were washed, harvested, lysed, and analyzed as described above.

Role of ATP Depletion in the Cellular Accumulation

HeLa cells were grown to near confluence in 6-well plates. On the day of the experiment, the culture media was removed, and the cells were treated with fresh media containing 50 mM 2-deoxy-D-glucose and 5 μM oligomycin A (<0.001% EtOH, originating from the oligomycin stock solution). Control cells were treated with an equivalent volume of ethanol. The cells were then incubated at 37 °C for 1 h before the complex (50 μM) was directly added to the well. The cells were incubated for another 3 h at 37 °C. The culture media was then removed, and the adherent cells were then washed, harvested, lysed, and analyzed as described above.

Role of Organic Cation Transporters (OCTs) in the Cellular Accumulation

HeLa cells were grown to near confluence in 6-well plates. On the day of the experiment, the culture media was removed, and the cells were treated with fresh media containing the OCT3 inhibitor 1,1′-diethyl-2,2′-cyanine iodide (decynium-22, 1 μM)[12,13] and the complex (50 μM). The cells were then incubated for 3 h. The culture media was then removed, and the adherent cells were washed, harvested, lysed, and analyzed as described above.

S7. CORTICAL NEURON ASSAYS

Cell Viability after OGD

Primary cortical neuron cultures were seeded at a density of 150,000 cells/well in 48-well plates for cell viability studies. After 10–12 days in culture, neurons were left untreated (vehicle) or treated with Os245 or Os245′ (1, 2.5, 5, 10, 30, or 50 μM) for 3 h before being subjected to 90 min of oxygen-glucose deprivation (OGD). For OGD, culture media was removed and 300 μL of glucose-free balanced salt solution (GBSS) containing corresponding concentrations of compound was added to wells. Culture plates were then placed in an airtight chamber flushed with a gas mixture of 90% nitrogen, 5% carbon dioxide and 5% hydrogen for 3 min to remove any oxygen from the chamber. The chamber was then placed in a 37 °C incubator for the remainder of the OGD treatment (90 min total). After the OGD treatment, the GBSS was removed, and 500 μL of fresh culture media containing corresponding concentrations of compound was added back to the wells. Cell viability was measured 24 h after OGD treatment using a 3-(4,5-dimethylthiazol-2-yl)-2,5-diphenyltetrazolium bromide (MTT) assay.

Assessment of Mitochondrial Function and Glycolysis

Primary cortical neuron cultures were seeded at a density of 100,000 cells/well in seahorse XF24 well plates for analysis of mitochondrial function using the seahorse XF24 extracellular flux analyzer. Seahorse assay analysis was performed on days 10–12 in culture. Neuron cultures were treated with Os245, Os245′, or left untreated (vehicle) for 3 h prior to being subjected to OGD for 30 min. Preparation of the calibration plates for sensor calibration was performed according to the manufacturer's protocol. After OGD, cultures were returned to fresh culture media for 1 h, and then the media was changed to an artificial cerebral spinal fluid containing: NaCl (120 mM), KCl (3.5 mM), $CaCl_2$ (1.3 mM), KH_2PO_4 (0.4 mM), $MgCl_2$ (1 mM), HEPES (20 mM), glucose (15 mM), sodium pyruvate (2 mM), and fatty acid free BSA (4 mg/ml). After 1 h equilibration in the media and proper calibration of plate sensors, the Seahorse XF extracellular flux assay was performed. Mitochondrial function was measured at baseline and after the sequential addition of oligomycin (2 μM), carbonyl cyanide-4-(trifluoromethoxy) phenylhydrazone (2 μM), rotenone (300 nM), and antimycin A (5 μM). Three measurements were acquired at each condition, providing an analysis of the oxygen consumption rate (OCR) and extracellular acidification rate (ECAR) of the neuron cultures.

S7. ANIMAL STUDIES

Measurement of seizure-like behaviors

Adult male C57/Bl6 mice (10–12 weeks old) were administered an intraperitoneal (i.p.) injection of 10 mg/kg of either Ru265, Os245, or Os245′ (n = 4 for each compound). Animals were then observed for over 2 h (135 min), and the duration of time each animal exhibited seizures was recorded.[14] Over time seizure activity changed from shorter (30–120 s) mild seizures to longer (8–10 min) more severe seizures. All mice were euthanized with an overdose injection of sodium pentobarbital (150 mg/kg, i.p.) 2.5 h after start of experiment.[14]

S9. Supplementary Figures and Tables

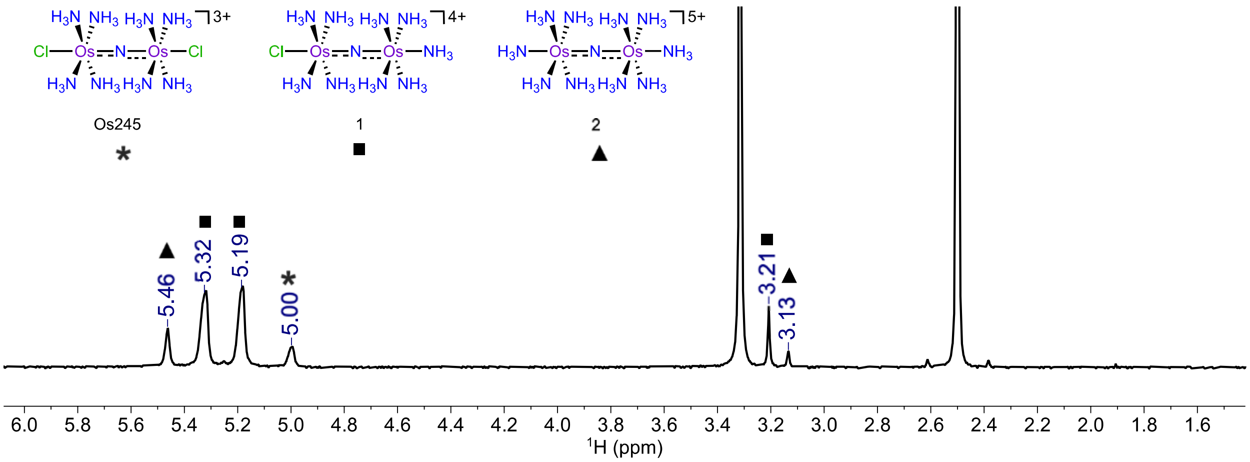

Figure S1. ^{1}H NMR spectra (500 MHz, 25 °C, DMSO-d_6) of the mixed product obtained from the reaction of $(NH_4)_2OsCl_6$ with conc. NH_4OH for 72 h.

Table S1. Summary of reaction conditions explored in the synthesis of Os245 and relative ratios of **1**, **2**, and Os245 obtained.[a]

Entry	Os Precursor	Temp (°C)	Time (h)	$[NH_4OH]$ (M)	Ratio **1**:**2**:Os245
1	$(NH_4)_2OsCl_6$	150	72	14	61:14:25[b]
2	$(NH_4)_2OsCl_6$	150	48	14	76:20:4
3	$(NH_4)_2OsCl_6$	150	12	14	82:4:14
4	K_2OsCl_6	150	72	14	83:11:6
5	K_2OsCl_6	110	48	14	69:4:27
6	K_2OsCl_6	110	72	7	79:4:17[b]
7	Na_2OsCl_6	110	72	14	68:6:26

a. relative ratio of products was determined by ^{1}H NMR spectroscopy of the crude product.
b. in some cases, additional unidentified minor products were observed under these conditions.

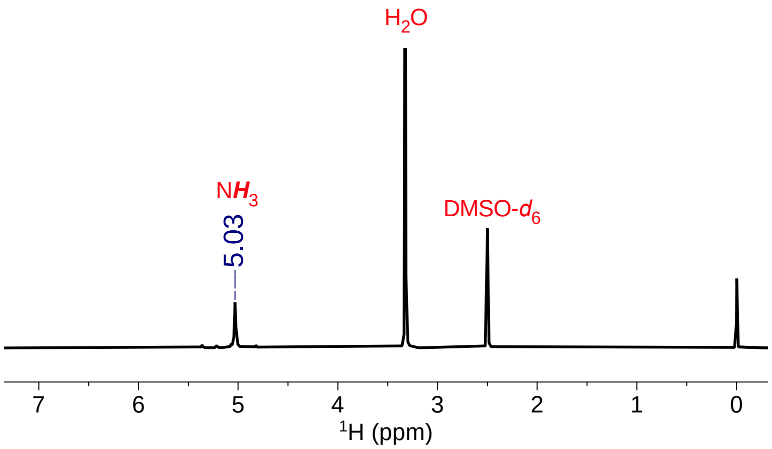

Figure S2. ^{1}H NMR (500 MHz, 25 °C) spectrum of Os245 in DMSO-d_6.

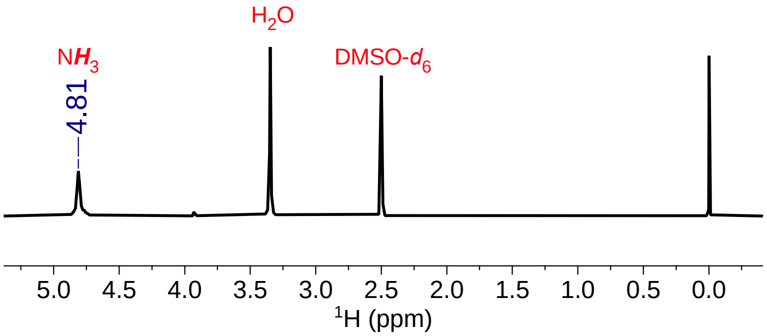

Figure S3. ^{1}H NMR (500 MHz, 25 °C) spectrum of Os245′ in DMSO-d_6.

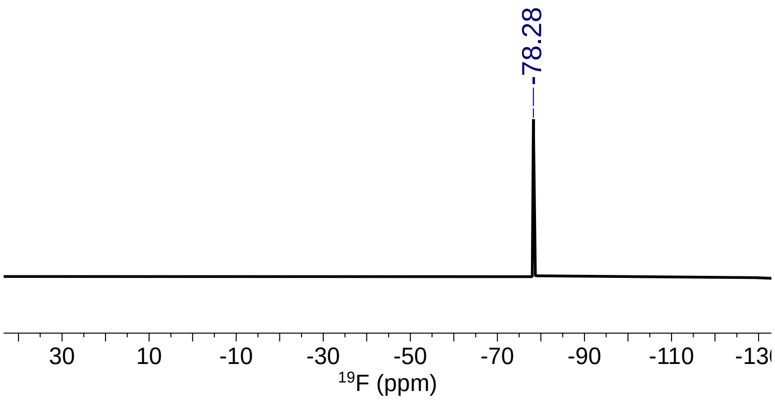

Figure S4. ^{19}F NMR (470 MHz, 25 °C) spectrum of Os245′ in DMSO-d_6.

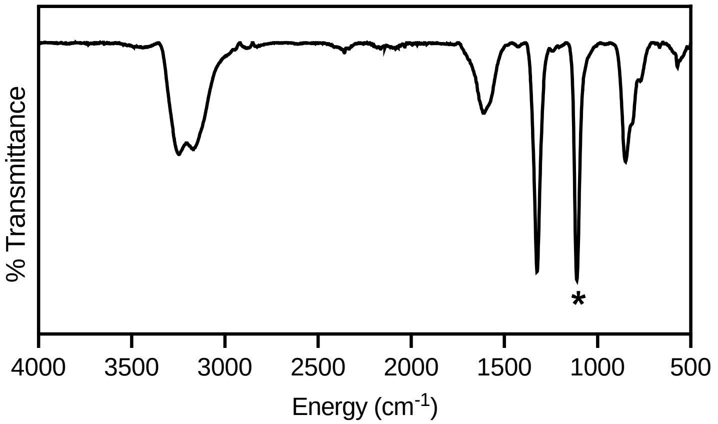

Figure S5. IR (ATR) spectrum of Os245. The asterisk indicates the band tentatively assigned asymmetric Os–N–Os stretching mode.

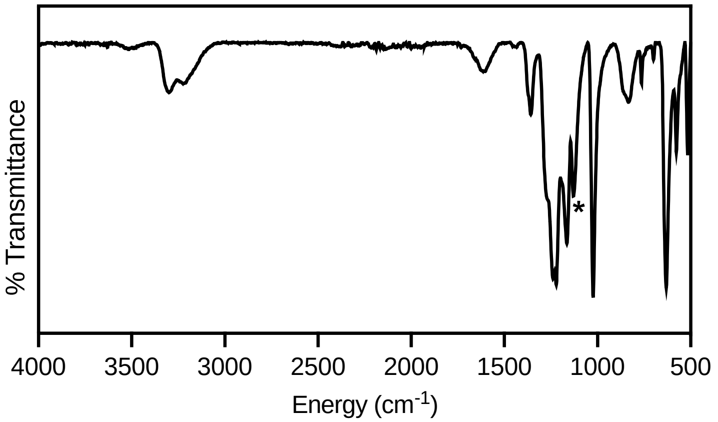

Figure S6. IR (ATR) spectrum of Os245′. The asterisk indicates the band tentatively assigned asymmetric Os–N–Os stretching mode.

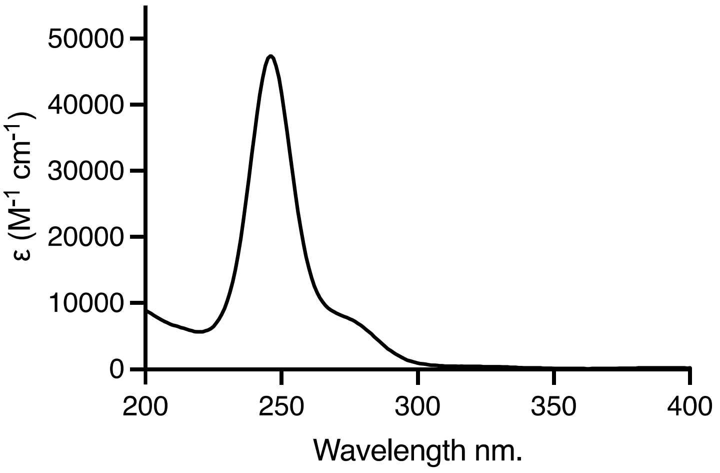

Figure S7. UV-vis spectrum of Os245 in 50 mM MOPS (pH 7.4) at 25 °C.

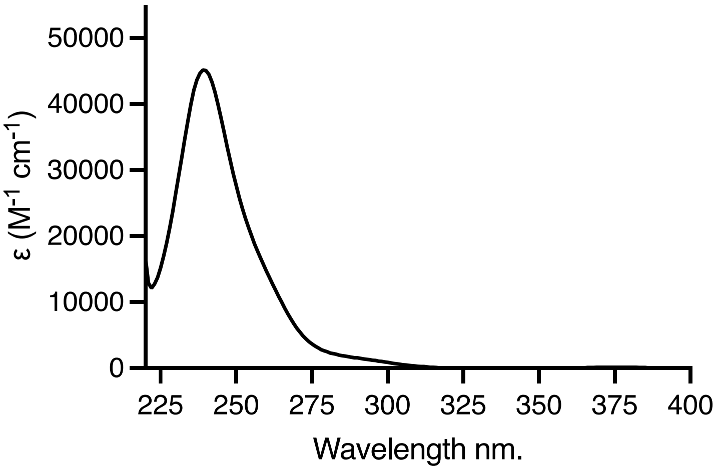

Figure S8. UV-vis spectrum of Os245′ in 50 mM MOPS (pH 7.4) at 25 °C.

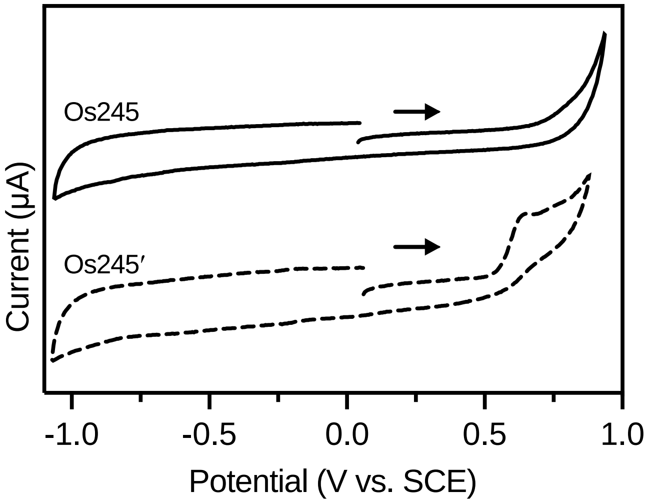

Figure S9. Cyclic voltammogram of (top) Os245 and (bottom) Os245′ in 0.1 M KCl buffered to pH 7.4 with 10 mM HEPES. Conditions: glassy carbon working electrode, Pt wire counter electrode, Ag/AgCl quasi-reference electrode as a 0.1 V s^{-1} scan rate. The scan was initiated at the resting potentials of the solutions (0.04 V for Os245 and 0.06 V for Os245′) and swept initially to anodic potentials. All potentials are referenced to the $[Ru(NH_3)_6]^{3+/2+}$ redox couple as an internal standard and are reported in V vs. SCE.

Table S2. Crystallographic parameters for the structures of Os245, Os245′, and **1**.

Compound	**Os245**	**Os245′**	**1**
Empirical Formula	$Cl_5H_{24}N_9OOs_2$	$H_{30}N_{14}O_{18}Os_2$	$C_3H_{36}Cl_2N_{10}O_{10}Os_2S_3$
Formula Weight	723.93	894.78	919.90
a (Å)	7.77840(10)	15.3326(2)	18.1826(5)
b (Å)	16.3909(2)	10.76340(10)	7.8130(2)
c (Å)	7.70290(10)	14.9970(2)	17.3198(5)
α(°)	90	90	90
β (°)	90	111.5530(10)	90
γ(°)	90	90	90
V (Å^3)	982.08(2)	2301.91(5)	2460.46(12)
Z	2	4	4
λ (Å)	0.71073	0.71073	0.71073
Crystal System	Orthorhombic	Monoclinic	Orthorhombic
Space Group	Cmmm	$P2_1/c$	Pnma
ρ_{calc} (Mg/m^3)	2.448	2.582	2.483
μ (mm^{-1})	13.607	11.135	10.848
T (K)	99.9(6)	99.9(5)	100.00(10)
2θ range (°)	2.485 – 31.604	2.390 – 31.363	2.240 – 25.248
Independent Reflections	912	6911	2403
R_{int}	0.0592	0.0550	0.0325
Number of Parameters	38	336	169
Largest diff. peak and hole (Å^{-3})	2.620 / -1.285	1.854 / -1.452	1.457 / -1.254
GoF	1.267	1.054	1.068
R1/wR2 (all data)	0.0279 / 0.0710	0.0237 / 0.0418	0.0238 / 0.0516
R1/wR2 (>2θ)	0.0264 / 0.0700	0.0207 / 0.0407	0.0205 / 0.0504

$R_1 = \Sigma||F_o|-|F_c||/\Sigma|F_o|$; $wR2 = \{\Sigma[w(F_o^2-F_c^2)^2]/\Sigma_w[(F_o^2)^2]\}^{1/2}$
GoF = $\{\Sigma[w(F_o^2-F_c^2)^2]/(n-p)\}^{1/2}$, where *n* is the number of data and *p* is the number of refined parameters.

Table S3. Selected interatomic distances (Å) and angles (°) for the crystal structure of Os245. Atoms are labeled as shown in Figure 2 of the main text.

Interatomic Distance (Å)	
Os–Cl(1)	2.438(2)[a]
Os–N(1)	1.7688(3)
Os–N(2)	2.123(4)

Interatomic Angle (°)	
Os–N(1)–Os#[b]	180.0
N(1)–Os–Cl(1)	180.0
N(1)–Os–N(2)	92.91(10)

a. Numbers given in parenthesis correspond to the standard error of the last digit of the interatomic distance or angle
b. # indicates symmetry generated atoms.

Table S4. Selected interatomic distances (Å) and angles (°) for the crystal structure of Os245′. Atoms are labeled as shown in Figure 2 of the main text.

Interatomic Distance (Å)			
Os(1)–O(1)	2.1348(18)[a]	Os(2)–O(2)	2.134(2)
Os(1)–N(1)	1.76340(9)	Os(2)–N(6)	1.76729(10)
Os(1)–N(2)	2.130(2)	Os(2)–N(7)	2.117(3)
Os(1)–N(3)	2.127(2)	Os(2)–N(8)	2.129(3)
Os(1)–N(4)	2.108(2)	Os(2)–N(9)	2.114(3)

Interatomic Angle (°)			
Os(1)–N(1)–Os(1)#[b]	180.0	Os(2)–N(6)–Os(2)#	180.0
N(1)–Os(1)–O(1)	178.50(5)	N(6)–Os(2)–O(2)	179.06(7)
N(1)–Os(1)–N(2)	96.03(6)	N(6)–Os(2)–N(7)	97.04(7)
N(1)–Os(1)–N(3)	93.81(6)	N(6)–Os(2)–N(8)	95.25(7)
N(1)–Os(1)–N(4)	92.65(6)	N(6)–Os(2)–N(9)	95.54(7)
N(1)–Os(1)–N(5)	93.78(6)	N(6)–Os(2)–N(10)	92.87(7)
N(2)–Os(1)–O(1)	84.92(8)	N(7)–Os(2)–O(2)	82.73(10)
N(3)–Os(1)–O(1)	85.03(8)	N(7)–Os(2)–N(8)	88.77(11)
N(3)–Os(1)–N(2)	89.91(9)	N(7)–Os(2)–N(10)	89.04(10)
N(4)–Os(1)–O(1)	86.42(8)	N(8)–Os(2)–O(2)	83.84(10)
N(4)–Os(1)–N(2)	171.29(9)	N(9)–Os(2)–O(2)	84.71(10)
N(4)–Os(1)–N(3)	90.27(9)	N(9)–Os(2)–N(7)	167.34(10)
N(4)–Os(1)–N(5)	88.68(9)	N(9)–Os(2)–N(8)	91.45(12)
N(5)–Os(1)–O(1)	87.37(8)	N(9)–Os(2)–N(10)	88.96(10)
N(5)–Os(1)–N(2)	90.00(9)	N(10)–Os(2)–O(2)	88.04(9)
N(5)–Os(1)–N(3)	172.37(9)	N(10)–Os(2)–N(8)	171.80(10

a. Numbers given in parenthesis correspond to the standard error of the last digit of the interatomic distance or angle

b. # indicates symmetry generated atoms.

Table S5. Selected interatomic distances (Å) and angles (°) for the crystal structure of **1**. Atoms are labeled as shown in Figure 2 of the main text.

Interatomic Distance (Å)			
Os(1)–Cl(1)	2.4357(13)[a]	Os(2)–N(2)	2.201(5)
Os(1)–N(1)	1.759(5)	Os(2)–N(1)	1.782(5)
Os(1)–N(5)	2.113(3)	Os(2)–N(3)	2.134(3)
Os(1)–N(6)	2.125(3)	Os(2)–N(4)	2.119(4)

Interatomic Angle (°)			
Os(1)–N(1)–Os(2)	176.1(3)	N(1)–Os(2)–N(2)	177.5(2)
N(1)–Os(1)–Cl(1)	178.39(16)	N(1)–Os(2)–N(3)	94.39(14)
N(1)–Os(1)–N(5)	94.16(15)	N(1)–Os(2)–N(4)	92.18(14)
N(1)–Os(1)–N(6)	91.98(14)	N(2)–Os(2)–N(3)	87.32(13)
N(5)–Os(1)–Cl(1)	86.98(9)	N(2)–Os(2)–N(4)	86.12(13)
N(6)–Os(1)–Cl(1)	86.90(9)		

a. Numbers given in parenthesis correspond to the standard error of the last digit of the interatomic distance or angle

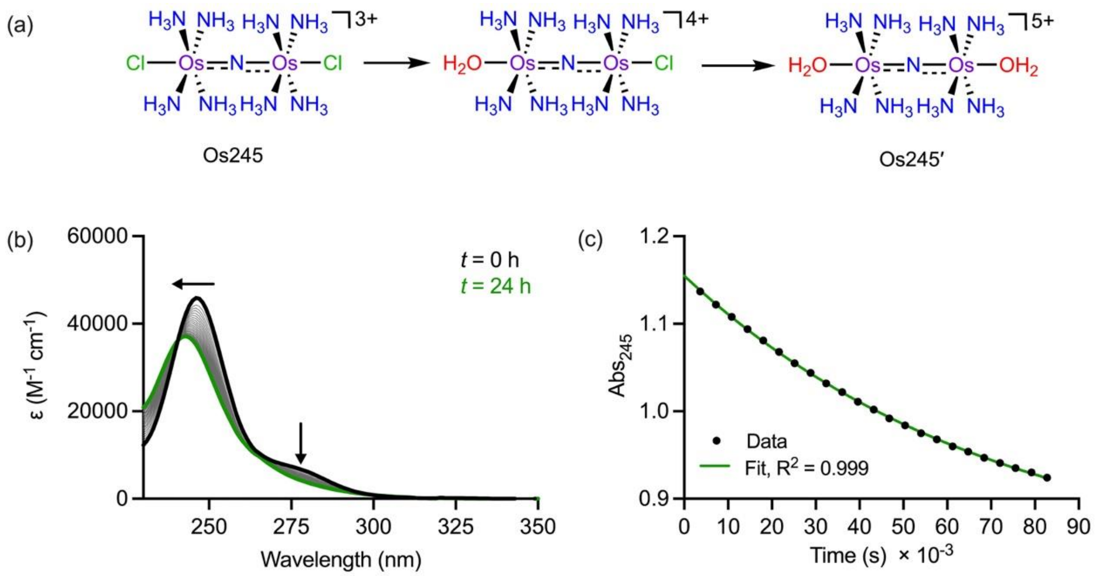

Figure S10. (a) Reaction scheme showing the expected aquation reaction of Os245. (b) Representative UV-vis spectral traces showing the aquation of Os245 in 50 mM MOPS buffer (pH 7.4) at 37 °C. Spectra were acquired every 1 h over 24 h. (c) Exponential fit of the absorbance at 245 nm to determine the pseudo-first order rate constant (k_{obs}, s^{-1}) for the aquation reaction shown in panel a.

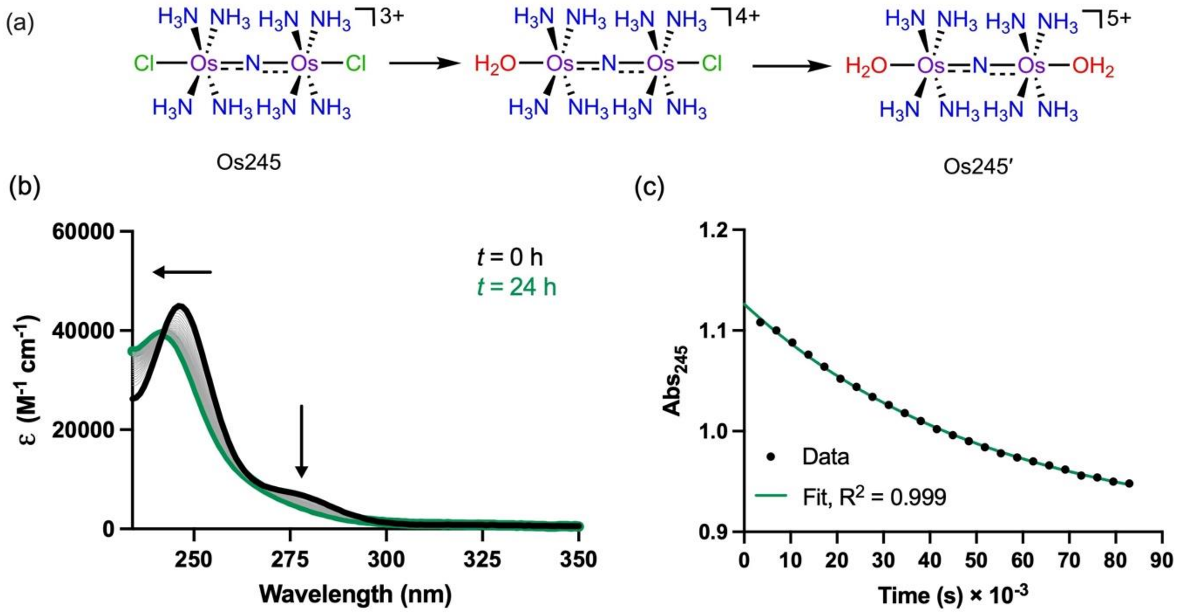

Figure S11. (a) Reaction scheme showing the expected aquation reaction of Os245. (b) Representative UV-vis spectral traces showing the aquation of Os245 in 50 mM MOPS buffer (pH 7.4) supplemented with 150 mM NaCl at 37 °C. Spectra were acquired every 1 h over 24 h. (c) Exponential fit of the absorbance at 245 nm to determine the pseudo-first order rate constant (k_{obs}, s^{-1}) for the aquation reaction shown in panel a.

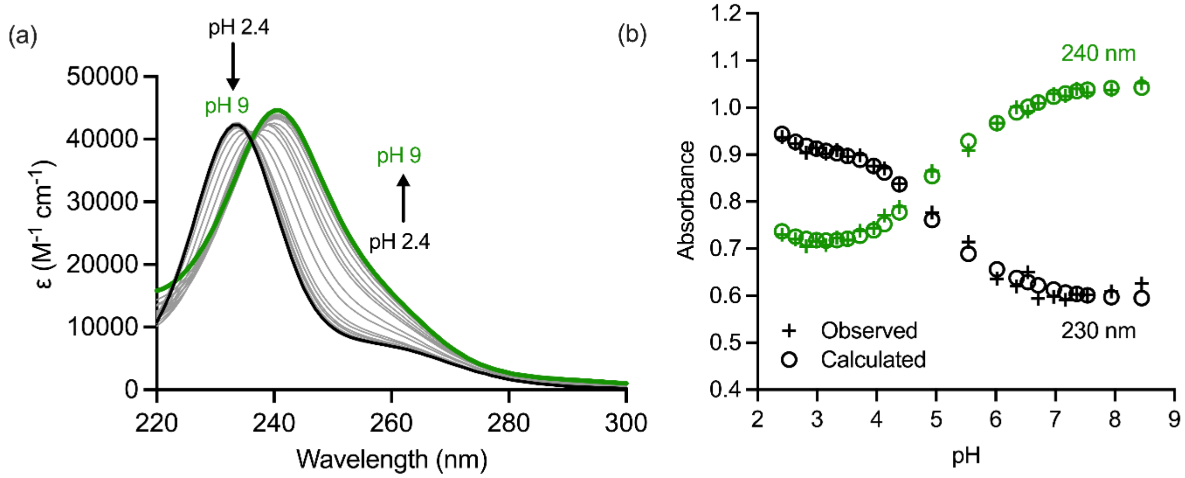

Figure S12. (a) Representative spectral changes of Os245′ over the pH range of 2.4 – 9 (25 °C, *I* = 0.1 M). (b) Representative data fitting at 230 and 240 nm over the pH range of 2.4 – 9. All spectra and absorbance values were corrected for the change in molar concentration of complex upon addition of titrant.

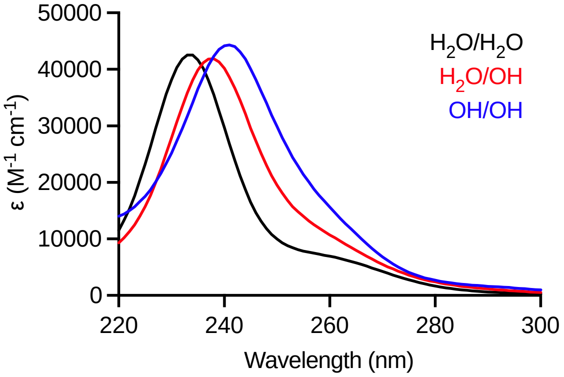

Figure S13. Predicted molar absorbance spectra of $[(H_2O)Os(NH_3)_4(\mu\text{-}N)Os(NH_3)_4(OH_2)]^{5+}$, $[(H_2O)Os(NH_3)_4(\mu\text{-}N)Os(NH_3)_4(OH)]^{4+}$, and $[(HO)Os(NH_3)_4(\mu\text{-}N)Os(NH_3)_4(OH)]^{3+}$ obtained from the data fitting procedure using HypSpec.

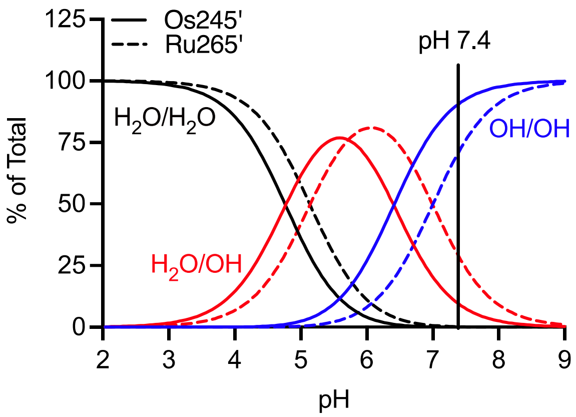

Figure S14. Comparison of the aqueous species distribution of Os245′ (solid lines) and Ru265′ (dashed lines) over the pH range of 2 – 9 (25 °C, *I* = 0.1 M). The protonation constants of Ru265′ were taken from Ref. 6. The vertical line indicates pH 7.4.

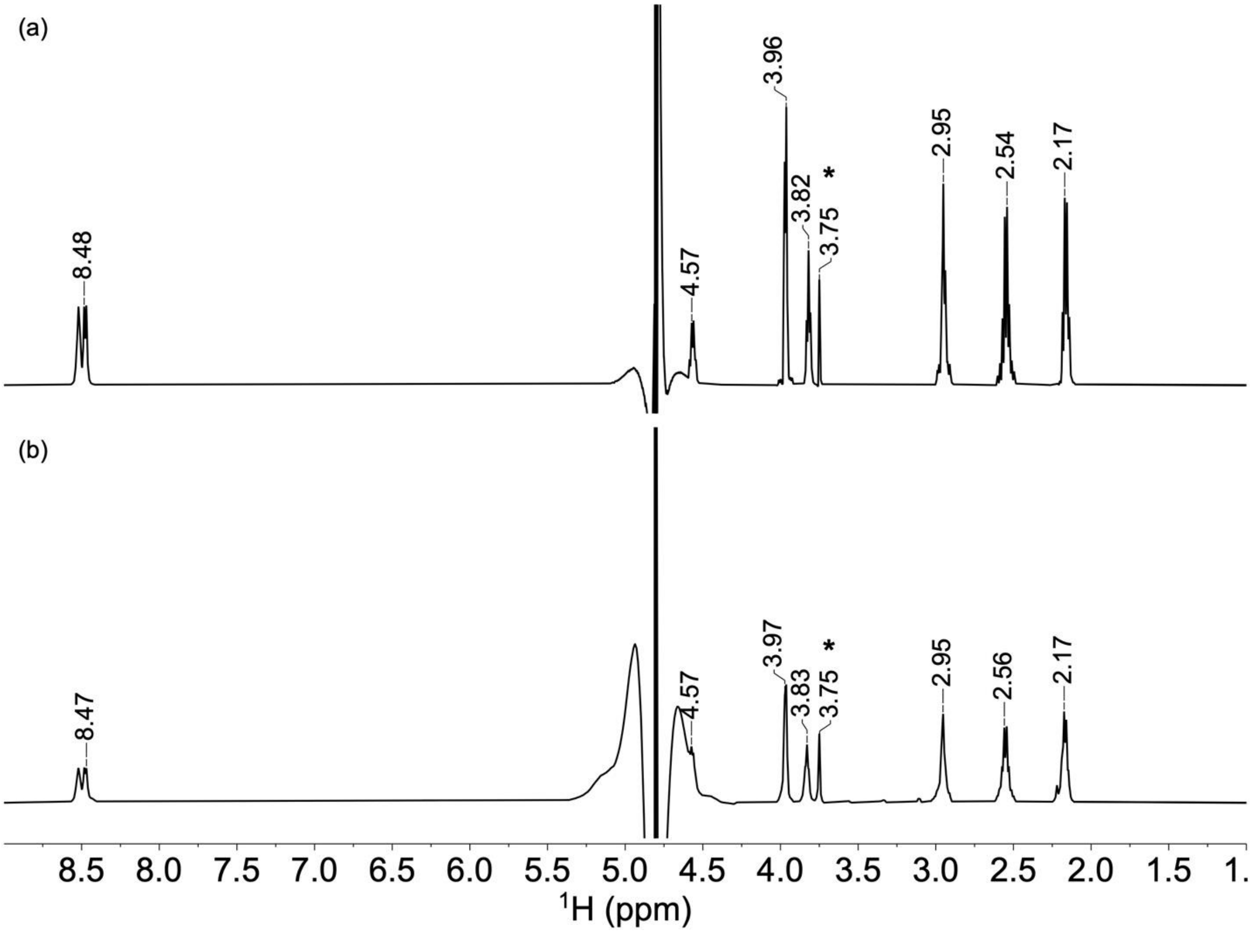

Figure S15. ^1H NMR spectra (500 MHz, 25 °C) with water suppression in 90% PBS/10% D_2O with < 1% dioxane reference, indicated by an asterisk (*), of (a) glutathione (GSH) and (b) 4-fold excess GSH incubated with Os245′ at 37 °C for 24 h. No significant binding between GSH and Os245 has been determined by the lack of spectral changes between (a) and (b).

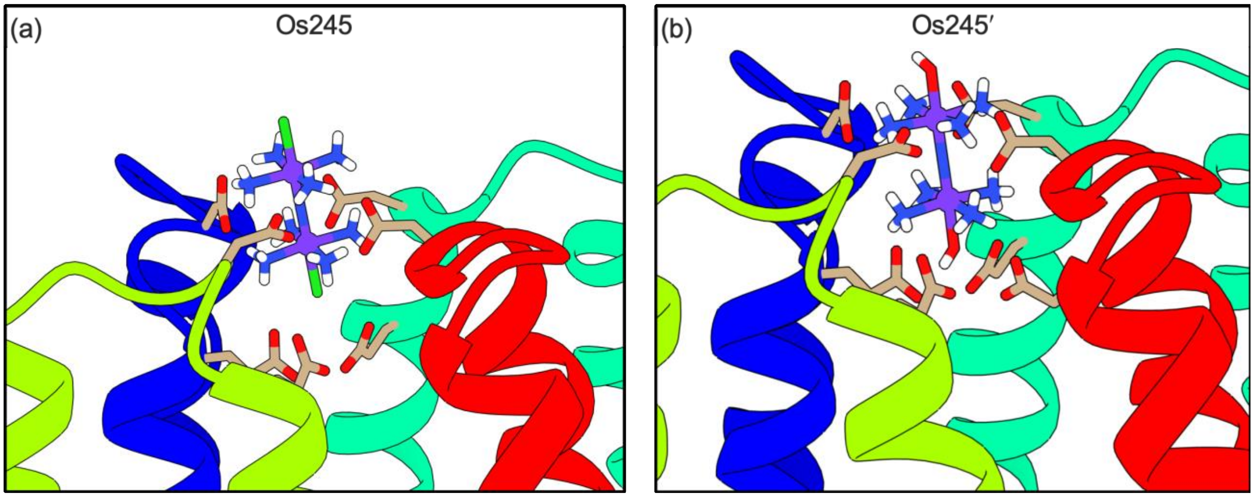

Figure S16. Molecular docking analysis of (a) Os245 and (b) Os245′ in the cytosolic pore of the MCU channel (PDB 6O5B). The symmetric hydroxido-capped form of Os245′ was used for all analyses as it is the major form of this complex at a physiologically relevant pH of 7.4. Atom colors: purple = Os, blue = N, red = O, white = H, tan = C.

Table S6. Comparison of the relative docking scores of the metal complexes docked into the DIME region of the human MCU (PDB 6O5B).

Compound	CHEMPLP Score
Os245	32.05
Os245′	47.26

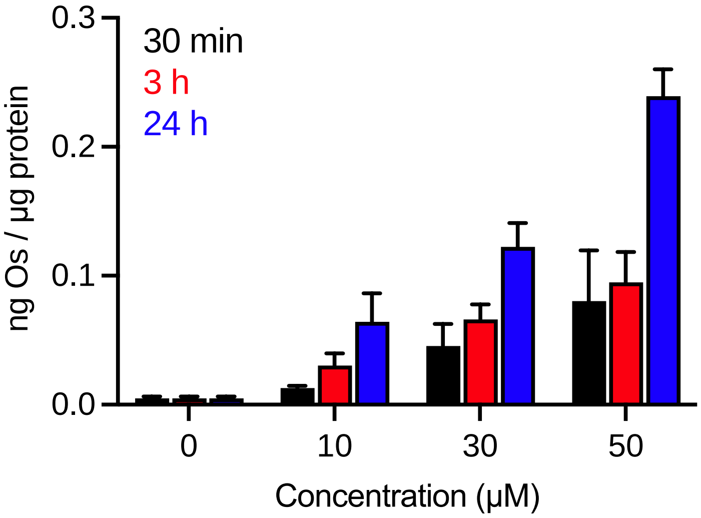

Figure S17. Cellular Os accumulation in primary cortical neurons treated with increasing concentrations of Os245 for 30 min, 3 h, or 24 h at 37 °C.

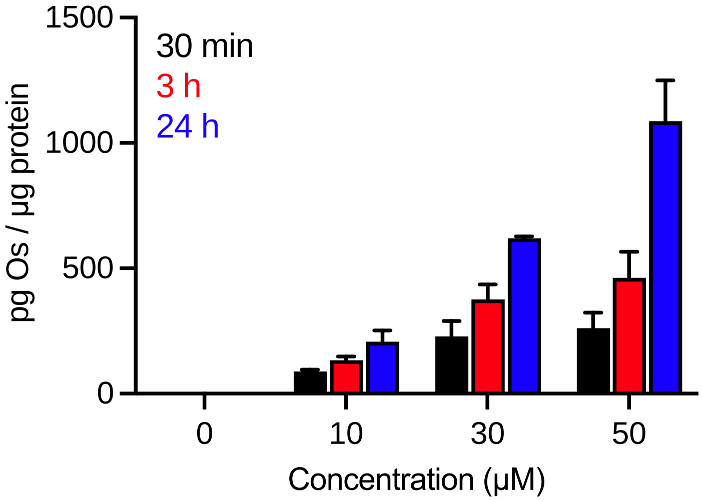

Figure S18. Cellular Os accumulation in primary cortical neurons treated with increasing concentrations of Os245′ for 30 min, 3 h, or 24 h at 37 °C.

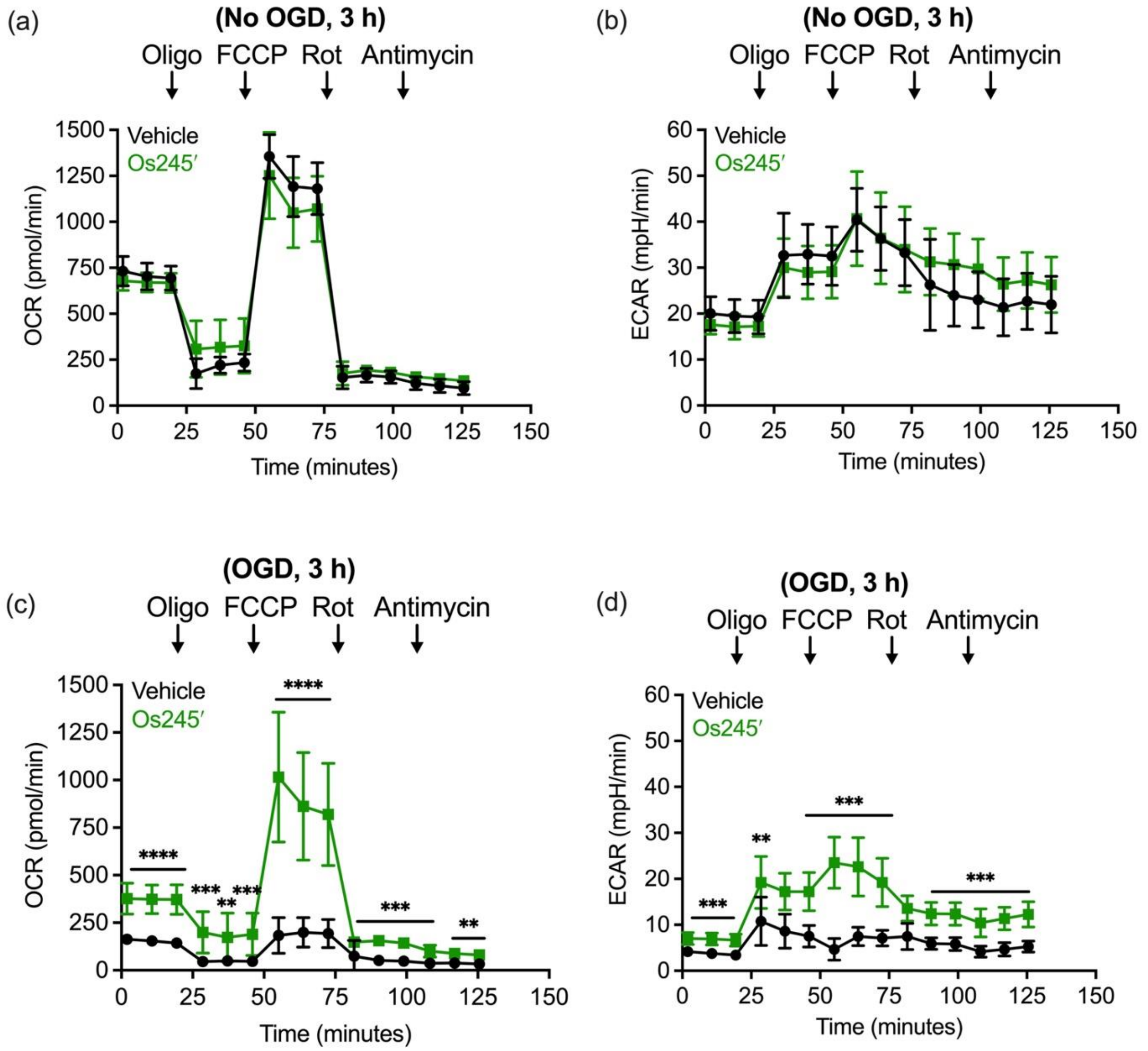

Figure S19. (a) Oxygen consumption rate (OCR) and (b) extracellular acidification rate (ECAR) measurements in primary cortical neuron cultures after the sequential addition of oligomycin (1 μM), FCCP (2 μM), rotenone (300 mM), and antimycin (1 μM) following treatment with Os245′ (75 μM) for 3 h. (c,d) Pretreatment with Os245′ (75 μM) for 3 h preserves (c) OCR and (d) ECAR in primary cortical neurons subjected to 30 min OGD.

S10. REFERENCES

(1) Vogt, L. H.; Katz, J. L.; Wiberley, S. E. *Inorg. Chem.* **1965**, *4*, 1157–1163.

(2) Klose, M. H. M.; Hejl, M.; Heffeter, P.; Jakupec, M. A.; Meier-Menches, S. M.; Berger, W.; Keppler, B. K. *Analyst* **2017**, *142*, 2327–2332.

(3) Sheldrick, G. M. *Acta Crystallogr. Sect. A Found. Crystallogr.* **2015**, *71*, 3–8.

(4) Sheldrick, G. M. *Acta Crystallogr. Sect. C Struct. Chem.* **2015**, *71*, 3–8.

(5) Müller, P. *Crystallogr. Rev.* **2009**, *15*, 57–83.

(6) Woods, J. J.; Lovett, J.; Lai, B.; Harris, H. H.; Wilson, J. J. *Angew. Chem. Int. Ed.* **2020**, *59*, 6482–6491.

(7) Gans, P.; O'sullivan, B. 2000; Vol. 51, pp 33–37.

(8) Korb, O.; Stützle, T.; Exner, T. E. *J. Chem. Inf. Model.* **2009**, *49*, 84–96.

(9) Cole, J. C.; Nissink, J. W. M.; Taylor, R. In *Virtual Screening in Drug Discovery*; Soichet, B., Alvarez, J., Eds.; Taylor and Francis CRC Press: Boca Raton, FL, 2005.

(10) Woods, J. J.; Rodriguez, M. X.; Tsai, C.-W.; Tsai, M.-F.; Wilson, J. J. *Chem. Commun.* **2021**, *57*, 6161–6164.

(11) Nichols, M.; Elustondo, P. A.; Warford, J.; Thirumaran, A.; Pavlov, E. V; Robertson, G. S. *J. Cereb. Blood Flow Metab.* **2017**, *37*, 3027–3041.

(12) Koepsell, H.; Lips, K.; Volk, C. *Pharm. Res.* **2007**, *24*, 1227–1251.

(13) Massmann, V.; Edemir, B.; Schlatter, E.; Al-Monajjed, R.; Harrach, S.; Klassen, P.; Holle, S. K.; Sindic, A.; Dobrivojevic, M.; Pavenstädt, H.; Ciarimboli, G. *Pflugers Arch. Eur. J. Physiol.* **2014**, *466*, 517–527.

(14) Novorolsky, R. J.; Nichols, M.; Kim, J. S.; Pavlov, E. V.; J Woods, J.; Wilson, J. J.; Robertson, G. S. *J. Cereb. Blood Flow Metab.* **2020**, *40*, 1172–1181.
